# Dosage-sensitive maternal siRNAs determine hybridization success in *Capsella*

**DOI:** 10.1038/s41477-024-01844-3

**Published:** 2024-11-11

**Authors:** Katarzyna Dziasek, Juan Santos-González, Kai Wang, Yichun Qiu, Jiali Zhu, Diana Rigola, Koen Nijbroek, Claudia Köhler

**Affiliations:** 1 Department of Plant Biology, Uppsala BioCenter, Swedish University of Agricultural Sciences and Linnean Centre for Plant Biology, Uppsala, Sweden; 2 Department of Plant Reproductive Biology and Epigenetics, Max Planck Institute of Molecular Plant Physiology, Potsdam, Germany; 3 Keygene N.V., Wageningen, the Netherlands

**Keywords:** Seed development, Plant hybridization, DNA methylation, Plant evolution

## Abstract

Hybrid seed failure arising from wide crosses between plant species is a recurring obstacle in plant breeding, impeding the transfer of desirable traits. This postzygotic reproductive barrier primarily occurs in the endosperm, a tissue that nourishes the embryo and functions similarly to the placenta in mammals. We found that incompatible seeds show a loss of DNA methylation and chromatin condensation in the endosperm, similar to seeds lacking maternal RNA polymerase IV activity. This similarity is linked to a decrease in small interfering RNAs in the endosperm (sirenRNAs), maternal RNA polymerase IV-dependent short interfering RNAs that regulate DNA methylation. Several AGAMOUS-like MADS-box transcription factor genes (*AGL*s), key regulators of endosperm development, are targeted by sirenRNAs in *cis* and in *trans*. This finding aligns with the enrichment of AGL target genes among deregulated genes. We propose that hybrid seed failure results from reduced maternal sirenRNAs combined with increased *AGL* expression, leading to abnormal gene regulation in the endosperm.

## Abstract (web summary)

Hybrid seed failure in plant breeding arises from disrupted endosperm development. Reduced maternal sirenRNAs and increased expression of AGL transcription factors cause abnormal gene regulation in the endosperm, preventing successful wide species crosses.

## Main

Hybrid seed failure resulting from wide crosses between plant species poses a recurring challenge in plant breeding, obstructing the transfer of desirable traits between species. This postzygotic reproductive barrier predominantly manifests in the endosperm—an embryo-nourishing tissue serving a similar function as the placenta in mammals^1^. In most flowering plants, the endosperm is triploid, comprising one paternal and two maternal genomes. This specific genomic ratio is pivotal for endosperm viability, as evidenced by seed abortion upon hybridization of plants with differing ploidy^2–4^. Much like failures in interploidy hybridization, crosses between closely related species with equal ploidy often result in seed arrest. The term ‘effective ploidy’ has been used to characterize this phenomenon, wherein certain species exhibit behaviour reminiscent of polyploid species, despite their actual diploid nature^5^. Remarkably, seeds arrested from both interploidy and interspecies crosses exhibit similar phenotypic and molecular abnormalities^6–8^, implying the existence of a dosage-sensitive element contributing to this reproductive barrier within the endosperm. However, the precise nature of this component remains elusive.

In the *Capsella* genus, interspecies hybridizations between the inbreeder *C. rubella* (*Cr*) and the outbreeder *C. grandiflora* (*Cg*) lead to a loss of chromatin condensation and DNA methylation in the endosperm, particularly in the CHG and CHH contexts (H represents all bases except G). This chromatin change is associated with an accumulation of deregulated genes in pericentromeric regions, many of which are targeted by type I AGAMOUS-like MADS-box transcription factors (AGLs)^9^. Type I AGLs belonging to the Mγ and Mγ-interacting Mα* clades are key regulators of endosperm proliferation and cellularization^10^. These data suggest that hypomethylation in the hybrid endosperm facilitates the access of AGLs to hypomethylated regions, resulting in detrimental gene activation. Similarly, in *Arabidopsis*, interploidy hybridizations of diploid (2x) maternal plants with tetraploid (4x) paternal plants causes endosperm hypomethylation in the CHG and CHH contexts^11^, which has been linked to a disruption of the RNA-directed DNA methylation (RdDM) pathway in the hybrid endosperm^12^. This pathway initiates with the plant-specific DNA-dependent RNA polymerase IV (Pol IV) generating short non-coding transcripts that are transformed into double-stranded RNA by RNA-DEPENDENT RNA POLYMERASE 2 (RDR2) and further processed into 24-nucleotide short interfering RNAs (siRNAs) by DICER-LIKE3. These siRNAs, when incorporated into ARGONAUTE (AGO) proteins, guide DNA methyltransferases to target sequences, inducing DNA methylation^13^. Notably, both ovules and endosperm accumulate highly abundant siRNAs over a modest number of distinct loci, commonly referred to as sirenRNAs (small interfering RNAs in the endosperm) that are primarily of maternal origin^14–16^. In *Arabidopsis*, sirenRNAs target *AGL*s, and their abundance is reduced in triploid seeds derived from crosses between 2x and 4x plants^17^. Nonetheless, whether sirenRNAs constitute the dosage-sensitive component determining hybridization success remains an open question.

Previous work revealed that the loss of paternal Pol IV function can suppress the interploidy hybridization barrier in *Arabidopsis*^11,14^. Interestingly, affected genes in the endosperm differ depending on whether the *nrpd1* mutation (disrupting Pol IV function) is maternally or paternally inherited, suggesting that maternal and paternal Pol IV-dependent siRNAs have distinct effects on endosperm development for reasons that remain to be fully elucidated^11,18^.

In this study, we demonstrate that the loss of maternal Pol IV function mirrors the loss of chromatin condensation and DNA methylation observed in *Capsella* hybrids. This resemblance in phenotype is correlated with a shared decrease in sirenRNAs, which regulate target genes in *cis* and *trans* by guiding DNA methylation. Among these sirenRNA targets are several *AGL*s, aligning with the enrichment of AGL target genes among deregulated genes. On the basis of these data, we propose that the response to interspecies and interploidy hybridizations is instigated by two factors: the depletion of maternally derived sirenRNAs and the heightened expression of *AGL*s targeting hypomethylated regions in hybrid endosperm.

## Results

### *Capsella* interploidy and interspecies hybrids share similar defects

**Fig. 1:**
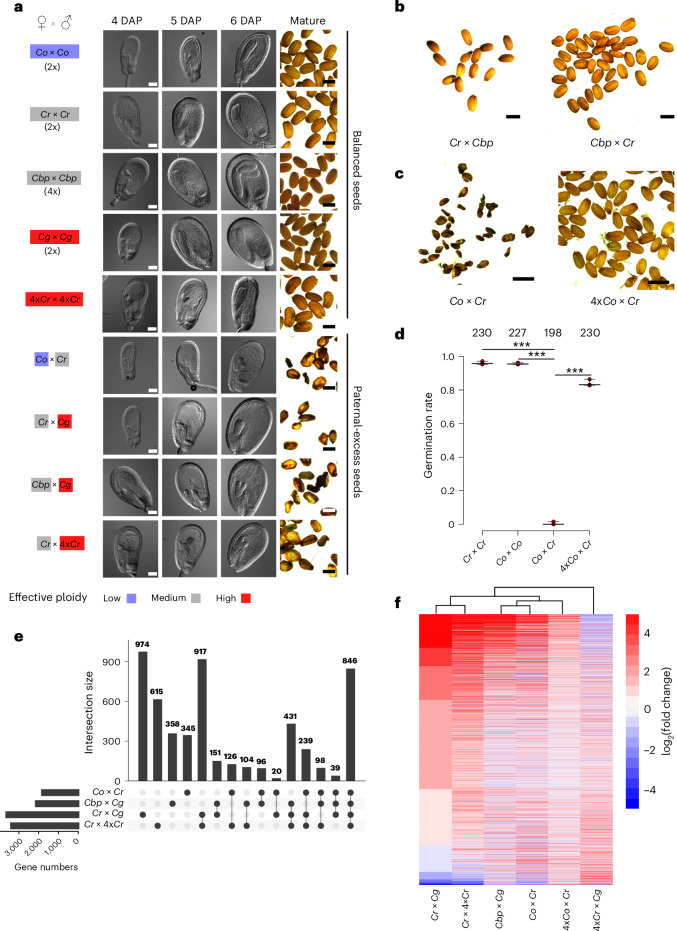
Interploidy and interspecies hybrid seeds share similar phenotypes and deregulated genes. **a**, Cleared hybrid *Capsella* seeds at 4 to 6 DAP. The corresponding mature seeds are shown on the right side of each panel. Scale bars, 100 µm and 1 mm for cleared and mature seeds, respectively. The colour code reflects effective ploidy as indicated in the figure. **b**, Mature seed phenotypes of crosses *Cr* × *Cbp* and *Cbp* × *Cr*. Scale bars, 1 mm. **c**, Mature seed phenotypes of crosses *Co* × *Cr* and 4x*Co* × *Cr*. Scale bars, 1 mm. **d**, Germination data for the crosses shown in **c**. Shown is the fraction of germinating seeds from each cross, with each filled circle representing one biological replicate; there are three biological replicates per genotype. The numbers at the top represent total seed numbers. The asterisks represent statistical significance as calculated by one-way analysis of variance with post-hoc Tukey’s honestly significant difference test (***P < 0.001). **e**, Upset plot showing the overlap of upregulated genes between interploidy (*Cr* × 4x*Cr*) and interspecies crosses of different *Capsella* species. **f**, Heat map and dendrogram show clustering of samples based on log_2_ fold changes (compared with the corresponding maternal parent) of genes upregulated in the analysed hybrids. Source data

**Extended Data Fig. 1:**
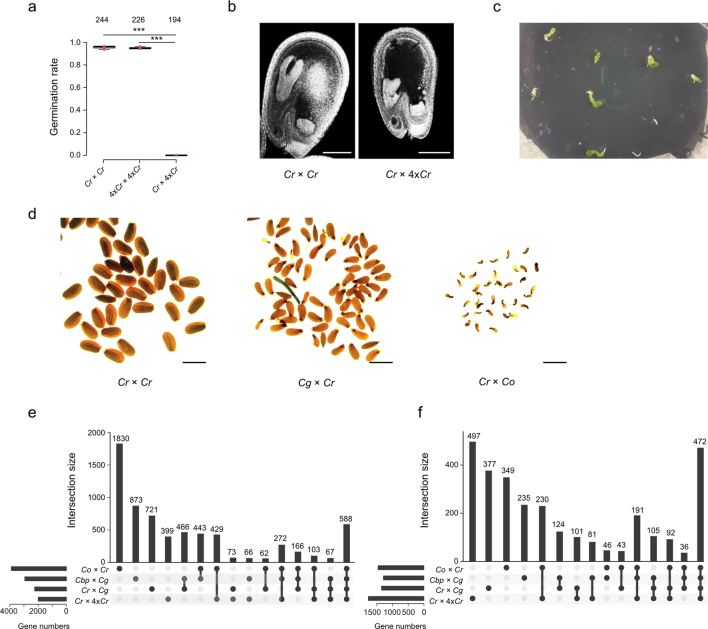
Effects of interploidy and interspecies crosses in *Capsella.* **a**, Germination of triploid *Cr* seeds shown as the fraction of germinating seeds from each cross. Each filled circle represents one biological replicate; there are three biological replicates per genotype. Numbers on top represent total seed number. Asterisks represent statistical significance as calculated by one way ANOVA with post hoc Tukey’s HSD test (*** p < 0.001). Red dots show values for three biological replicates. Whiskers show the full range excluding outliers. **b**, Feulgen staining of diploid and triploid *Cr* seeds at 6 DAP (scale bars represent 200 µm). **c**, *Cr* × 4x*Cr* embryos rescued on Murashige-Skoog medium. **d**, Seed phenotypes of *Cr* × *Cr, Cg* × *Cr* and *Cr* × *Co* seeds. Scale bars represent 1 mm. **e**, **f**. Upset plot showing the overlap of upregulated genes in comparison to the paternal parent (**e**) and both parents (**f**) between interploidy (*Cr* × 4x*Cr*) and interspecies crosses of different *Capsella* species. Source data

To test whether interploidy and interspecies hybridization share a common molecular basis, we analysed the cross between 2x maternal and 4x paternal *Cr* plants (referred to as *Cr* × 4x*Cr*; by convention the female parent is always mentioned first). As previously reported for paternal-excess interploidy crosses in *Arabidopsis*^3^, the resulting 3x*Cr* seeds were dark and shrivelled and failed to germinate (Fig. 1a and Extended Data Fig. 1a; the term ‘paternal-excess’ is used to refer to crosses where the paternal parent has higher numerical or effective ploidy than the maternal parent). The defects of 3x*Cr* seeds were related to failed endosperm cellularization (Extended Data Fig. 1b), resembling previously reported defects of interspecies paternal-excess hybrid seeds in *Capsella*, *Arabidopsis* and other species^3,6,19,20^. As in the case of interspecific hybrids^21^, 3x embryos could be rescued by removing them from the seeds and growing them in vitro (Extended Data Fig. 1c), supporting the view that the failure of 3x embryo survival is a consequence of a defect in endosperm development.

To determine whether phenotypic similarities in interploidy and interspecies hybrids are reflected by a similar molecular signature, we generated transcriptome data of hybrid seeds (six days after pollination (DAP)) resulting from incompatible crosses. These included *Cr* × 4x*Cr* interploidy crosses and previously characterized paternal-excess interspecies crosses of *Cr* × *Cg* and *C. orientalis* (*Co*) × *Cr*^21^ with the corresponding intraspecific crosses. We furthermore included the cross of *C. bursa-pastoris* (*Cbp*) × *Cg* (Supplementary Data 1). Despite *Cbp* being an allotetraploid species, when used as the maternal parent in crosses with *Cg*, *Cbp* exhibited strikingly similar behaviour to *Cr*. The resultant seeds exhibited a paternal-excess phenotype, characterized by shrinkage and abortion, with embryos arrested at the torpedo stage (Fig. 1a). Correspondingly, reciprocal crosses of *Cr* × *Cbp* gave rise to normal, viable seeds (Fig. 1b), revealing that *Cbp*, despite being a tetraploid species, behaved like a diploid. On the basis of the cross-incompatibilities between the species, we assigned an effective ploidy order of *Co* < *Cr* = *Cbp* < *Cg* = 4x*Cr*, consistent with previously published work^21^. Using the parent with higher effective ploidy as the maternal parent gave rise to maternal-excess phenotypes^21^ (Extended Data Fig. 1d), supporting the assigned order of effective ploidy. In line with theoretical predictions^22^, this order aligned with the breeding mode of the species: while *Cg* is an outbreeder, *Cr*, *Co* and *Cbp* are inbreeding species. However, whereas *Co* became an inbreeder as early as ~900,000 years ago, *Cr* and *Cbp* made the transition to inbreeding only ~200,000 years ago^23,24^. Previous work revealed that increased ploidy of the maternal *Cr* parent can restore the viability of hybrid *Cr* × *Cg* seeds^21^. Similarly, we found that increased ploidy of the maternal *Co* parent could suppress *Co* × *Cr* hybrid seed defects. While seeds derived from *Co* × *Cr* crosses aborted with similar morphological and transcriptional defects as *Cr* × *Cg*, *Cbp* × *Cg* and *Cr* × 4x*Cr* hybrids^21^ (Fig. 1a,c,d), seeds obtained from crosses of 4x*Co* × *Cr* were phenotypically like non-hybrid seeds and able to germinate (Fig. 1c,d).

We identified deregulated genes in the hybrids by comparing the hybrid transcriptome with that of the corresponding maternal parent (Fig. 1e), the paternal parent and both parents (Extended Data Fig. 1e,f). Our data revealed that all tested hybrids shared a large set of commonly upregulated genes, indicative of a common genetic basis. To test the molecular consequences of increased maternal ploidy, we profiled the transcriptomes of 4x*Cr* × *Cg* and 4x*Co* × *Cr* seeds and found that most genes that were upregulated in *Cr* × *Cg* and *Co* × *Cr* hybrid seeds were either downregulated or unchanged in hybrid seeds derived from a 4x maternal plant (Fig. 1f). These data support the idea that a dosage-sensitive component generated by the maternal parent determines hybridization success. Together, these data strongly suggest that interploidy and interspecies hybridization barriers share a common molecular basis. Moreover, the fact that increased ploidy of the maternal parent can alleviate paternal-excess hybrid incompatibility implies the existence of a quantitative maternal factor determining hybridization success.

### Incompatible hybrids lose chromatin condensation and CHG and CHH methylation

**Fig. 2:**
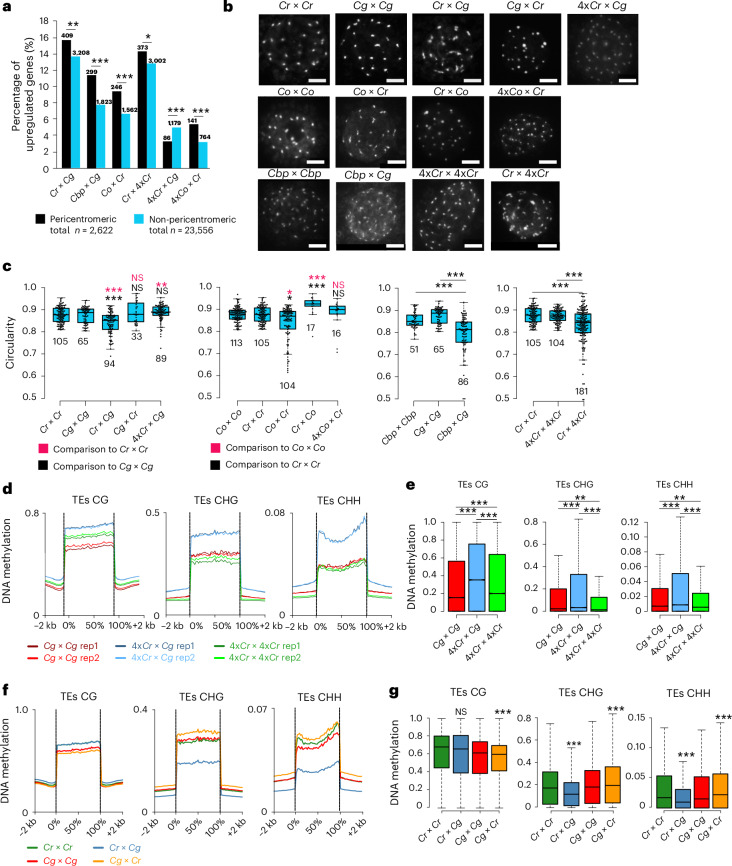
Interspecies hybridization causes chromatin condensation defects in endosperm nuclei. **a**, Percentage of genes in pericentromeric and non-pericentromeric regions that are upregulated in different incompatible *Capsella* hybrids. The numbers above the bars represent numbers of upregulated genes in pericentromeric and non-pericentromeric regions of a respective genotype. The asterisks represent statistical significance as calculated by two-sided chi-squared tests; *P* values were adjusted for multiple comparisons with Benjamini and Hochberg correction (***P < 0.001; **P < 0.01; *P < 0.05). **b**, DAPI-stained chromocenters from endosperm nuclei at 4 DAP of different *Capsella* genotypes. Scale bars, 5 μm. **c**, Quantification of chromatin condensation given as mean circularity of chromocenters (Methods). The numbers below the boxes indicate the number of analysed nuclei. The asterisks represent statistical significance as calculated by two-sided Wilcoxon tests; *P* values were adjusted for multiple comparisons with Benjamini and Hochberg correction (***P < 0.001; **P < 0.01; *P < 0.05). NS, not significant. **d**, Metagene plots showing DNA methylation levels of TEs in the endosperm of *Cg* × *Cg*, 4x*Cr* × *Cg* and 4x*Cr* × 4x*Cr* 6 DAP seeds. **e**, Box plots showing methylation levels of TEs in the endosperm of 6 DAP seeds of the indicated genotypes. The asterisks indicate statistically significant differences as calculated by two-sided Wilcoxon tests; *P* values were adjusted for multiple comparisons with Benjamini and Hochberg correction (***P < 0.001; **P < 0.01). **f**, Metagene plots showing DNA methylation levels of TEs in the endosperm of *Cg* × *Cr* 6 DAP compared with published data for *Cr* × *Cr*, *Cg* × *Cg* and *Cr* × *Cg* (ref. ^9^). **g**, Box plots showing methylation levels of TEs in the endosperm of 6 DAP seeds of the indicated genotypes. The asterisks indicate statistically significant differences as calculated by two-sided Wilcoxon tests; *P* values were adjusted for multiple comparisons with Benjamini and Hochberg correction (***P < 0.001). In **c**,**e**,**g**, the horizontal lines show the median values, the box edges show the interquartile range and the whiskers show the full range excluding outliers. Source data

Previous work revealed that *Cr* × *Cg* hybrid endosperm has decondensed chromocenters, which relates to a preferential expression of genes localized in pericentromeric regions^9^. To test whether this phenomenon is a general pattern connected with hybrid incompatibility in the *Capsella* genus, we analysed the locations of upregulated genes in paternal-excess crosses in relation to their positions on the chromosomes. Strikingly, we found that in all interspecies and interploidy paternal-excess hybrids, there was a preferential location of upregulated genes close to pericentromeric regions (Fig. 2a). We tested whether this phenomenon corresponded to a loss of chromosome condensation and analysed chromocenter condensation in a range of paternal-excess incompatible hybrids. Consistent with the similar molecular phenotypes of interspecies and interploidy paternal-excess hybrid seeds, we found that all hybrid nuclei had significantly reduced chromocenter condensation compared with non-hybrid nuclei (Fig. 2b,c and Supplementary Figs. 1 and 2). Importantly, chromocenter condensation defects and gene deregulation were suppressed in the nuclei of 4x*Cr* × *Cg* and 4x*Co* × *Cr* hybrids, suggesting that a dosage-sensitive maternal factor regulates chromatin condensation in the endosperm (Fig. 2a–c). While there were substantially fewer genes deregulated in the 4x*Co* × *Cr* than in the *Co* × *Cr* hybrids, deregulated genes remained enriched in pericentromeric regions (Fig. 2a). This suggests that despite visibly restored chromatin condensation upon increased maternal ploidy (Fig. 2c), these regions remained more accessible than in the wild type.

**Extended Data Fig. 2:**
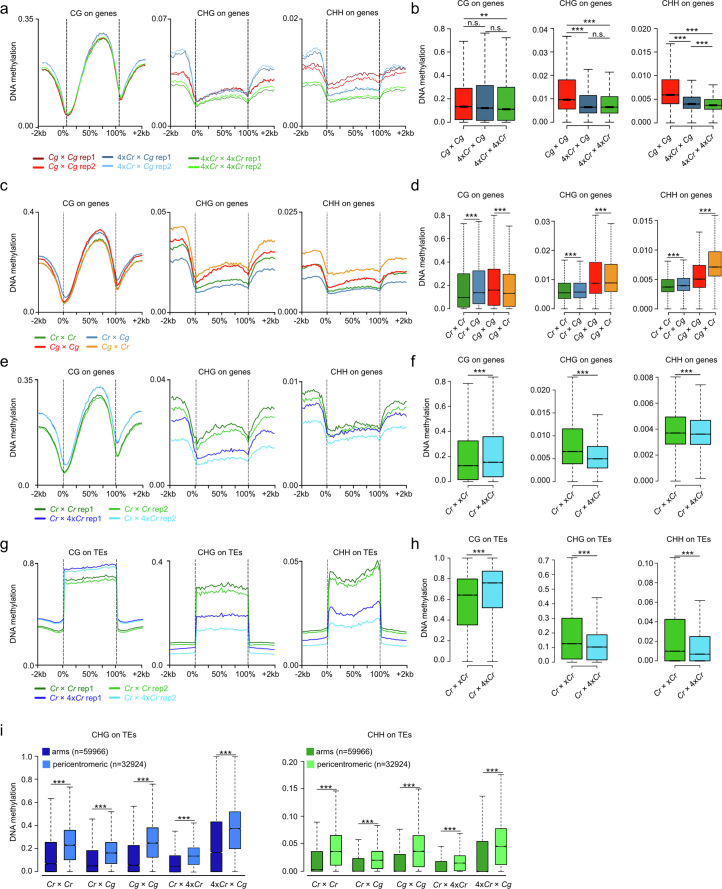
DNA methylation in the endosperm of different *Capsella Cr* and *Cg* hybrid crosses. **a**. Metagene plots show DNA methylation levels of genes in the endosperm of *Cg* × *Cg*, 4x*Cr* × *Cg* and 4x*Cr* × *Cr* 6 DAP seeds. **b**, Boxplots show methylation level of genes in the endosperm of *Cg* × *Cg*, 4x*Cr* × *Cg* and 4x*Cr* × *Cr* 6 DAP seeds. Asterisks indicate statistically significant differences as calculated by the two-sided Wilcoxon test, p-values were adjusted for multiple comparisons with Benjamini and Hochberg correction (** p-value <0.01,*** p-value < 0.001, n.s. – not significant). **c**, Metagene plots show DNA methylation levels of genes in the endosperm of *Cg* × *Cr* at 6 DAP in comparison to published data for *Cr* × *Cr, Cg* × *Cg, Cr* × *Cg*^*1*^. **d**, Boxplots show methylation level of genes in the endosperm *Cg* × *Cr* at 6 DAP in comparison to published data for *Cr* × *Cr, Cg* × *Cg, Cr* × *Cg*^*9*^. Asterisks indicate statistically significant differences as calculated by the two-sided Wilcoxon test, p-values were adjusted for multiple comparisons with Benjamini and Hochberg correction (*** p-value < 0.001). **e**, **f**, Metagene (**e**) and box plots (**f**) show DNA m2ethylation levels of genes in the endosperm of diploid and triploid 6 DAP *Cr* seeds. Asterisks indicate statistically significant differences as calculated by the two-sided Wilcoxon test, p-values were adjusted for multiple comparisons with Benjamini and Hochberg correction (*** p-value < 0.001). **g**, **h**, Metagene (**g**) and box plots (**h**) show DNA methylation levels of TEs in the endosperm of diploid and triploid 6 DAP *Cr* seeds. Asterisks indicate statistically significant differences as calculated by the two-sided Wilcoxon test, p-values were adjusted for multiple comparisons with Benjamini and Hochberg correction (*** p-value < 0.001). **i**. Boxplots show CHG (left panel) and CHH (right panel) methylation levels on TEs in chromosome arms and pericentromeric regions in the endosperm of *Cr* × *Cr*, *Cr* × *Cg* and *Cg* × *Cg* and *Cr* × 4x*Cr* at 6 DAP. Asterisks indicate statistically significant differences as calculated by the two-sided Wilcoxon test (*** p-value < 0.001). **b**, **d**, **f**, **h**, **i**, Boxes show median values and the interquartile range. Whiskers show the full range excluding outliers. Source data

**Extended Data Fig. 3:**
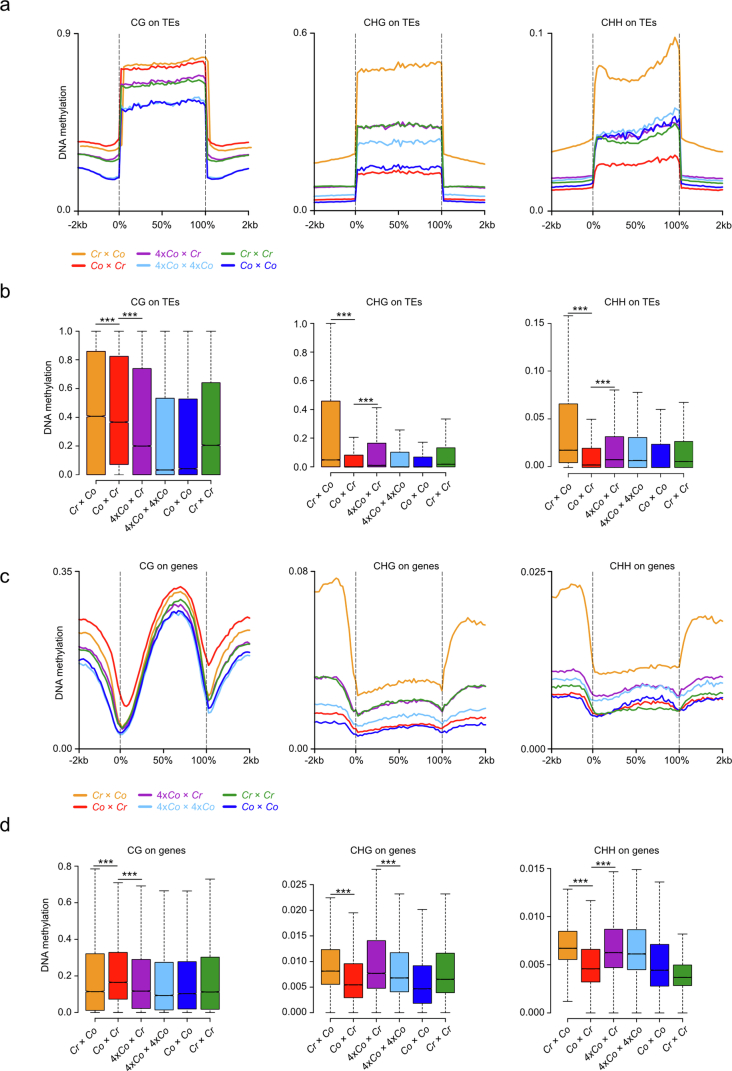
DNA methylation in the endosperm of different *Capsella Co* and *Cr* hybrid crosses. **a**, **b**, Metagene (**a**) and box plots (**b**) show DNA methylation levels of TEs in the endosperm of *Cr* × *Co*, *Co* × *Cr*, 4x*Co* × *Cr*, 4x*Co* × 4x*Co*, *Co* × *Co* and *Cr* × *Cr* at 6 DAP. Asterisks indicate statistically significant differences as calculated by the two-sided Wilcoxon test (*** p-value < 0.001). **c**, **d**, Metagene (**c**) and box plots (**d**) show DNA methylation levels of TEs in the endosperm of *Cr* × *Co*, *Co* × *Cr*, 4x*Co* × *Cr*, 4x*Co* × 4x*Co*, *Co* × *Co* and *Cr* × *Cr* at 6 DAP. Asterisks indicate statistically significant differences as calculated by the two-sided Wilcoxon test (*** p-value < 0.001). **b**, **d**, Boxes show median values and the interquartile range. Whiskers show the full range excluding outliers. Source data

Decondensation of pericentromeric regions in *Cr* × *Cg* and *Co* × *Cr* hybrid endosperm co-occurred with a loss of CHG and CHH methylation^9^, which was reversed by increasing the maternal ploidy (4x*Cr* × *Cg* and 4x*Co* × *Cr*) and in the reciprocal maternal-excess *Cg* × *Cr* and *Cr* × *Co* hybrids (Fig. 2d–g and Extended Data Figs. 2a–d and 3). Similarly, the loss of chromatin condensation in the nuclei of interploidy hybrids was related to reduced CHG and CHH methylation on genes and transposable elements (TEs) (Extended Data Fig. 2e–h). The loss of DNA methylation on TEs was most prominent in pericentromeric regions (Extended Data Fig. 2i), consistent with preferential upregulation of genes in this region^9^. Together, these data reveal that interploidy and interspecies hybridization cause similar molecular defects consistent with the similar phenotypes of hybrid seeds.

### Maternal *nrpd1* causes loss of chromatin condensation and CHG and CHH methylation

**Fig. 3:**
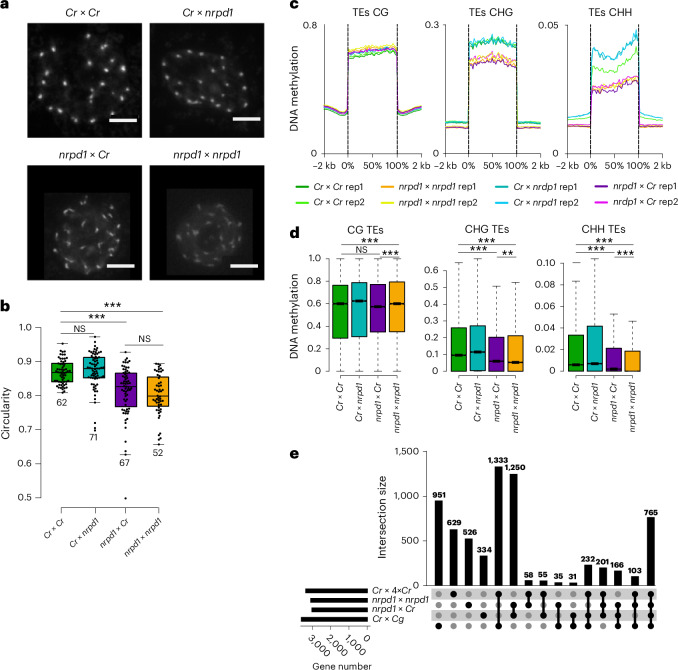
Maternal loss of *NRPD1* mimics paternal-excess hybrid phenotypes. **a**, DAPI-stained chromocenters from endosperm nuclei from *Cr* × *Cr*, *Cr* × *nrpd1*, *nrpd1* × *Cr* and *nrpd1* × *nrpd1*. Scale bars, 5 μm. **b**, Quantification of chromatin condensation given as mean circularity of chromocenters (Methods). The numbers below the boxes indicate the number of analysed nuclei. The asterisks represent statistical significance as calculated by two-sided Wilcoxon tests; *P* values were adjusted for multiple comparisons with Benjamini and Hochberg correction (***P < 0.001). **c**, Metagene plots showing DNA methylation levels of TEs in the endosperm of *Cr* × *Cr*, *nrpd1* × *nrpd1*, *Cr* × *nrpd1* and *nrpd1* × *Cr* 6 DAP seeds. **d**, Box plots showing methylation levels of TEs in the endosperm of 6 DAP seeds of the indicated genotypes. The asterisks indicate statistically significant differences as calculated by two-sided Wilcoxon tests; *P* values were adjusted for multiple comparisons with Benjamini and Hochberg correction (***P < 0.001; **P < 0.01). In **b** and **d**, the horizontal lines show the median values, the box edges show the interquartile range and the whiskers show the full range excluding outliers. **e**, Upset plot showing the overlap of upregulated genes among *Cr* × 4x*Cr*, *nrpd1* × *nrpd1*, *nrpd1* × *Cr* and *Cr* × *Cg* derived seeds. Genes were considered as upregulated on the basis of log_2_(fold change) > 1 and *P*_adj_ < 0.05 compared with *Cr* × *Cr*. Source data

**Extended Data Fig. 4:**
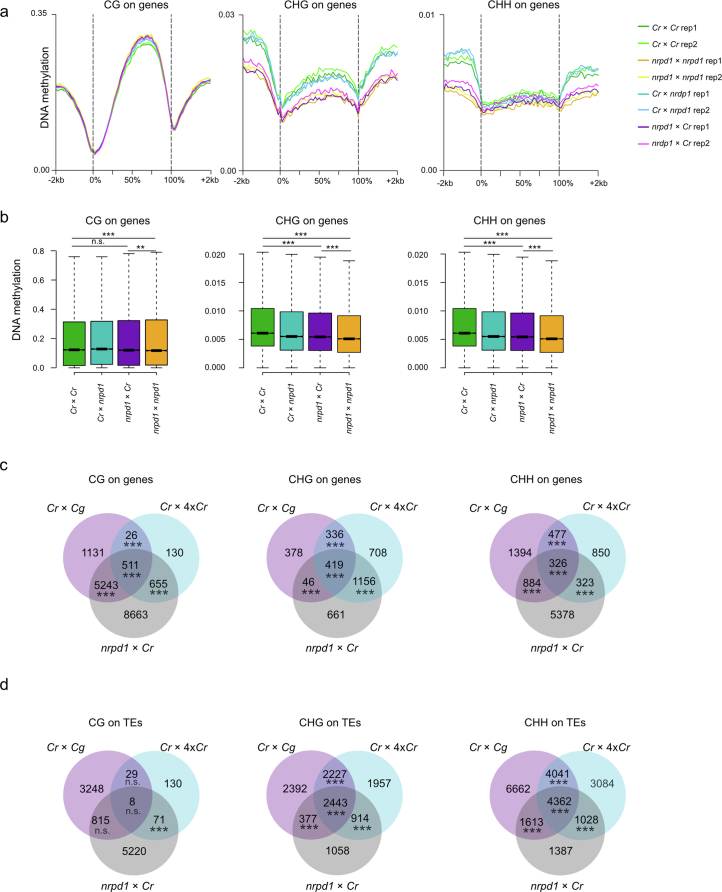
Maternal loss of *NRPD1* mimics DNA methylation changes seen in paternal-excess hybrids. **a**, Metagene plots show DNA methylation levels of genes in the endosperm of *Cr* × *Cr*, *nrpd1* × *nrpd1*, *Cr* × *nrpd1*, and *nrpd1* × *Cr* 6 DAP seeds. **b**, Boxplots show methylation level of genes in the endosperm at 6 DAP of the indicated genotypes. Boxes show median values and the interquartile range. Whiskers show the full range excluding outliers. Asterisks indicate statistically significant differences as calculated by the two-sided Wilcoxon test, p-values were adjusted for multiple comparisons with Benjamini and Hochberg correction (*** p-value < 0.001, ** pvalue < 0.01, * p-value < 0.05). **c**, **d**, Venn diagrams show the overlap of genes (**c**) and TEs (**d**) losing DNA methylation in the endosperm derived from *Cr* × *Cg*, *Cr* × 4x*Cr* and *nrpd1* × *Cr* crosses. Asterisks indicate statistically significant differences calculated using the supertest function from R package SuperExactTest (*** p-value < 0.001)^70^. Source data

**Extended Data Fig. 5:**
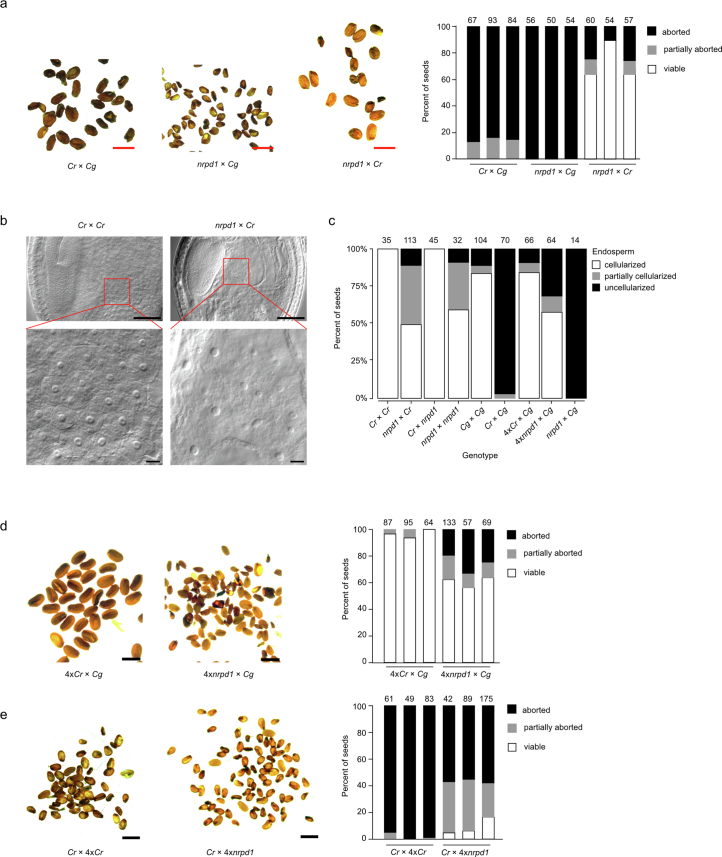
Seed and endosperm phenotypes in reciprocal crosses with *nrpd1.* **a**, Seed phenotypes of *Cr* × *Cg, nrpd1* × *Cg* and *nrpd1* × *Cr* seeds. Scale bar represents 1 mm. Barplot shows number of aborted and partially aborted seeds of three biological replicates of each genotype. Numbers above the bars indicate number of analysed seeds. **b**, Pictures of cleared seeds of *Cr* × *Cr* and *nrpd1* × *Cr* at 6 DAP. Inset shows enlarged regions of cellularized (*Cr* × *Cr*) and uncellularized (*nrpd1* × *Cr*) endosperm. Scale bars represents 100 µm (upper row) and 10 µm (lower row). **c**, Quantification of endosperm cellularization of indicated genotypes at 6 DAP. Numbers correspond to numbers of analysed seeds. **d**, Seed phenotypes of 4x*Cr* × *Cg* and 4x*nrpd1* × *Cg* seeds. Scale bar represents 1 mm. Barplot shows number of aborted and partially aborted and viable seeds of three biological replicates of each genotype. Numbers above the bars indicate number of analysed seeds. **e**, Seed phenotypes of *Cr* × 4x*Cr* and Cr × 4x*nrpd1* seeds. Scale bar represents 1 mm. Barplot shows number of aborted and partially aborted and viable seeds of three biological replicates of each genotype. Numbers above the bars indicate number of analysed seeds. Source data

The observed reduction of CHG and CHH methylation upon interploidy and interspecies hybridization^9^ (Fig. 2d–g and Extended Data Figs. 2 and 3) suggests a role of the RdDM pathway in establishing hybridization barriers in the endosperm. Pol IV is required to produce precursor RNAs, which are converted into 24-nucleotide siRNAs that guide de novo DNA methylation in all sequence contexts^13^. We thus tested whether a mutant in *NRPD1*, encoding the largest subunit of Pol IV, would cause a similar effect on chromatin condensation and the loss of DNA methylation. Indeed, we found that maternal loss of *NRPD1* reduced chromatin condensation in the endosperm, while the loss of paternal *NRPD1* had no effect, and the loss of maternal and paternal NRPD1 function did not enhance chromatin decondensation (Fig. 3a,b). Chromatin decondensation upon the loss of maternal NRPD1 function was associated with the loss of CHG and CHH methylation on genes and TEs in the endosperm, with similar regions being affected in interploidy and interspecies hybrids and in seeds depleted of maternal NRPD1 function (Fig. 3c,d and Extended Data Fig. 4a–d). This was also reflected by a large set of similarly deregulated genes in the endosperm of seeds lacking maternal NRPD1 function and that of interploidy and interspecies hybrids (Fig. 3e and Supplementary Data 1). Consistent with the similar molecular phenotype, endosperm cellularization was delayed in seeds derived from *nrpd1* mutant maternal plants, leading to a 10–20% abortion rate (Extended Data Fig. 5a–c). Maternal *nrpd1* also caused aggravated seed phenotypes in *nrpd1* × *Cg* crosses and seed abortion in 4x*nrpd1* × *Cg* crosses (Extended Data Fig. 5a,c,d). Previous studies found no evidence of *NRPD1* being imprinted in *Capsella*^21,25^, suggesting that maternal Pol IV-dependent siRNAs are generated in maternal sporophytic tissues, consistent with previous work^16,26^. Together, we concluded that reduced dosage of maternal Pol IV-dependent siRNAs affects DNA methylation, chromatin condensation and gene expression in a similar manner as interploidy and interspecies hybridizations. These data raise the hypothesis that interploidy and interspecies hybrid endosperms are depleted of maternal Pol IV-dependent siRNAs, causing loss of DNA methylation, decondensation of pericentromeric regions and activation of genes. Similar to previous findings^11,14^, we found that paternal loss of NRPD1 function could suppress interploidy seed arrest (Extended Data Fig. 7e), supporting the idea that maternal and paternal Pol IV-dependent siRNAs have different functions.

### Maternal sirenRNAs are depleted in hybrid endosperm

**Fig. 4:**
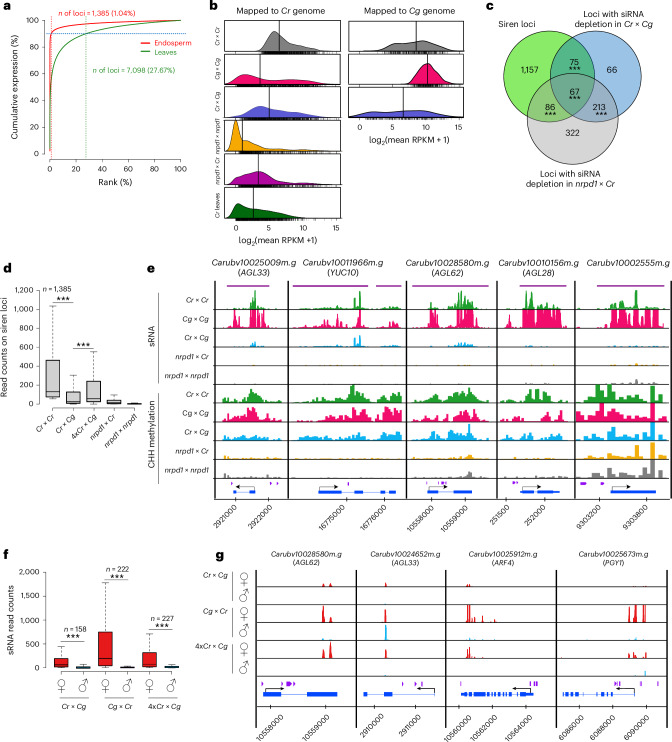
Maternal sirenRNAs are depleted in hybrid endosperm. **a**, Cumulative expression plot of 18–25-nucleotide siRNA loci in endosperm and leaves. Only loci with expression ≥2 reads per million (RPM) in combined replicates were analysed (*n* = 13,616 in endosperm and 13,495 in leaves). In endosperm and leaves, 0.99% and 27.6% of the most highly expressed 24-nucleotide-dominant loci account for 90% of siRNAs, respectively. **b**, Mean siRNA accumulation at siren loci in the endosperm of the indicated genotypes and *Cr* leaves. siRNA abundance at each locus is normalized by locus length (in kb) and library size. The mean of two replicates is presented. The black vertical lines represent medians. Individual measurements are shown as the rug below each density. The left plots show data obtained by mapping reads to the *Cr* genome; the right plots show data obtained by mapping reads to the *Cg* genome. RPKM, reads per kilobase million. **c**, Venn diagram showing the overlap among siren loci, loci with siRNA depletion in the *Cr* × *Cg* hybrid and loci with siRNA depletion in *nrpd1* × *Cr* (log_2_(fold change) < 0, *P*_adj_ < 0.1). The significance of overlap was calculated using the supertest function from the SuperExactTest package in R^70^ (***P < 0.001). **d**, Box plots showing the accumulation of siRNAs (18–25 nucleotides) on siren loci in different genotypes. The asterisks indicate statistically significant differences as calculated by two-sided Wilcoxon tests (***P < 0.001). **e**, siRNA accumulation and CHH methylation in the endosperm of the indicated genotypes on selected genes overlapping siren loci (marked with violet lines). The blue boxes represent genes; the purple boxes represent TEs. **f**, Parental-specific accumulation of sRNAs on loci having at least 15 parental-specific reads in total. The numbers above the boxes indicate the number of such genes in each genotype. The asterisks indicate statistically significant differences as calculated by two-sided Wilcoxon tests (***P <0.001). In **d** and **f**, the horizontal lines show the median values, the box edges show the interquartile range and the whiskers show the full range excluding outliers. **g**, Parental-specific sRNA accumulation in the endosperm of the indicated genotypes on selected genes. The blue boxes represent genes; the purple boxes represent TEs. Source data

**Extended Data Fig. 6:**
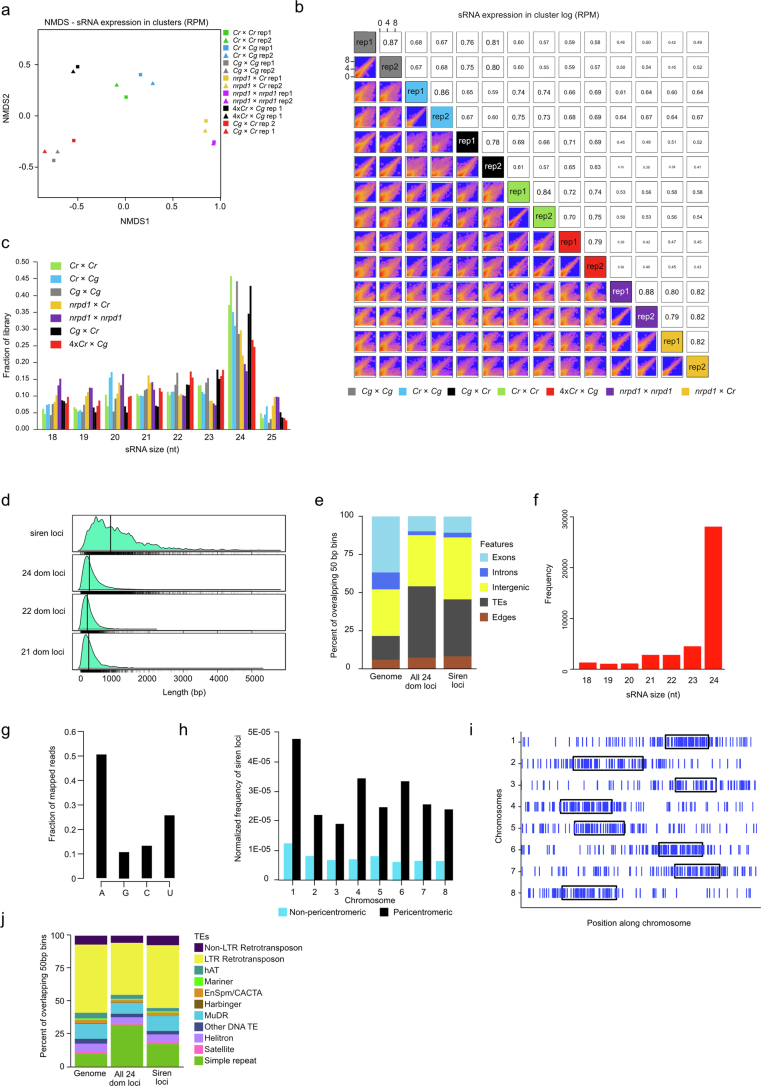
Quality of small RNA-Seq data and characterization of sirenRNAs. **a**, Non-metric MultiDimensional Scaling (NMDS) of small RNA (sRNA) sequencing samples. **b**, Correlogram showing the correlation score matrix across all sequenced sRNA libraries. **c**, Barplot shows size distribution of sRNAs in all sequenced libraries. **d**, Length distribution of siRNA loci. Siren loci are larger than other siRNA expressing loci, including non-siren 24 nt-dominant, 21 nt-dominant and 22 nt-dominant loci. **e**, Genomic features overlapping with 24 nt-dominant clusters (n=28 075) and siren loci (n=1 385) in *Cr*. The genome was subdivided into 50-bp windows (n= 2 497 626) that were assigned to either exons, introns, intergenic regions, transposable elements and edges (50bp regions at the boundaries of gene bodies and repeats). **f**, Frequency distribution of sirenRNA clusters after dominating size class. 24 nt siRNAs dominate in the majority of sirenRNA clusters defined by ShortStack. **g**, 5′ nucleotide of sirenRNAs (18–24 nt) in *Cr* × *Cr* endosperm. **h**, Number of siren loci normalized per size of respective genomic regions (in bp) – non-pericentromeric and pericentromeric for each of the eight *Cr* chromosomes. **i**, Distribution of siren loci along the eight chromosomes of the *Cr* genome. Blue bars indicate siren loci, black boxes indicate positions of pericentromeric regions. **j**, TE families overlapping with24 nt-dominant and siren loci compared to the genome-wide frequencies in *Cr*. Source data

**Extended Data Fig. 7:**
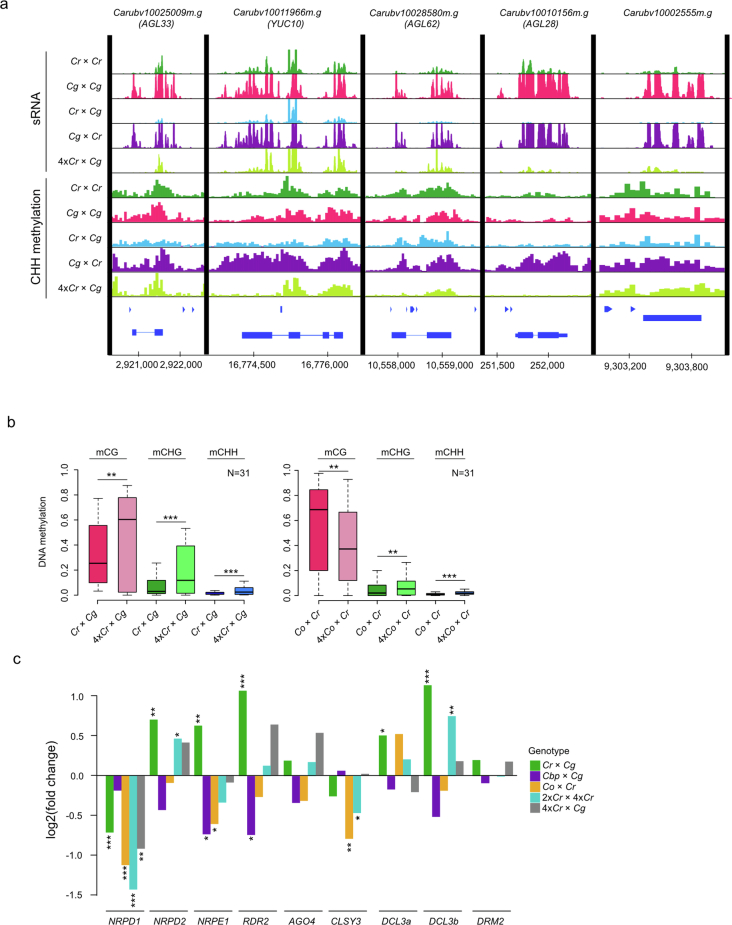
sirenRNA accumulation and DNA methylation of selected *AGLs* and expression of RdDM pathway genes. **a**, siRNA accumulation and CHH methylation in the endosperm of indicated genotypes on selected genes overlapping siren loci: *Carubv10025009m.g* (*AGL33*), *Carubv10011966m.g* (*YUC10*), *Carubv10028580m.g* (*AGL62*), *Carubv10010156m.g* (*AGL28*), and *Carubv10002555m.g* (γ-type AGL). Blue boxes represent genes, purple boxes represent TEs. **b**, Boxplots show DNA methylation on γ and α* type I MADS box TFs in 6 DAP endosperm of different *Capsella* genotypes. Boxes show median values and the interquartile range. Whiskers show the full range excluding outliers. Asterisks indicate statistically significant differences as calculated by the two-sided Wilcoxon test for paired samples, p-values were adjusted with Benjamini and Hochberg correction (*** p-value < 0.001, ** p-value < 0.01, * p-value < 0.05). **c**, Barplot shows log2 fold change expression level of indicated genes in comparison to respective maternal plants. Asterisks indicates statistical significance as calculated with Wald test in DESEQ2 (*** padj < 0.001, ** p- padj < 0.01, padj < 0.05). Source data

To understand the role of maternal *NRPD1* in hybrid incompatibility, we sequenced small RNAs (sRNAs) in manually dissected endosperm of 6 DAP seeds derived from crosses of *Cr* × *Cr*, *Cg* × *Cg*, *Cr* × *Cg*, *Cg* × *Cr*, 4x*Cr* × *Cg*, *nrpd1* × *Cr* and *nrpd1* × *nrpd1* (Supplementary Table 1). Profiles of sRNAs correlated well among biological replicates of the same genotype but were clearly distinct between genotypes (Extended Data Fig. 6a,b). Consistent with previous work^14^, we found that 24-nucleotide siRNAs were the predominant siRNA species in the endosperm (Extended Data Fig. 6c). Using ShortStack^27^, we identified 13,616 clusters accumulating 24-nucleotide siRNAs in the endosperm of *Cr* × *Cr* seeds. Of those clusters, only a few loci produced high levels of siRNAs in *Cr* × *Cr* endosperm, resembling the characteristics of siren loci^15,16^ (Fig. 4a and Supplementary Data 2). We identified 1,385 loci giving rise to 90% of the total siRNAs in *Cr* × *Cr* endosperm, which we will refer to as siren loci. As previously reported^16^, siren loci were longer than other loci expressing siRNAs (Extended Data Fig. 6d) and overlapped TEs, intergenic regions and genes (Extended Data Fig. 6e). SirenRNA loci were enriched for 24-nucleotide siRNAs (Extended Data Fig. 6f) with an adenine bias at the 5′ nucleotide (Extended Data Fig. 6g), suggesting interaction with AGO4-related AGO proteins^28^, consistent with previous observations in *Brassica*^16^. SirenRNA loci are distributed over the length of the chromosomes with an enrichment in pericentromeric regions (Extended Data Fig. 6h,i), but without a pronounced preference for any specific TE family (Extended Data Fig. 6j). We tested the percentage of maternally produced sirenRNAs by analysing the number of siren loci having reduced siRNAs in the *nrpd1* *×* *Cr* cross. We found that out of 1,385 sirenRNA loci, 985 (71.12%) had at least twofold reduced siRNAs. Similarly, in the *nrpd1* × *nrpd1* endosperm, 1,220 siren loci (88.09%) were depleted by at least twofold (Fig. 4b–d). There are conflicting data on the origin of sirenRNAs; while they have been proposed to be predominantly maternally produced in *Brassica*^16,29^, biparental production was proposed in *Arabidopsis*^18^. Our data suggest that most likely both the maternal seed coat and the endosperm are the source of sirenRNAs in the endosperm, with a predominant fraction being maternally derived. Like in maternal *nrpd1* endosperm, in *Cr* × *Cg* hybrid endosperm sirenRNA abundance was strongly depleted (Fig. 4b–d). Nearly all loci losing siRNAs in *Cr* × *Cg* endosperm corresponded to siren loci (Fig. 4c,d), strongly suggesting that maternally produced sirenRNAs are the dosage-sensitive component that becomes depleted in hybrid endosperm. Consistent with this idea, increased maternal genome dosage restored sirenRNAs in 4x*Cr* × *Cg* hybrids (Fig. 4d). To further test the parental origin of sirenRNAs, we made a parental-specific analysis of our sRNA data and identified several loci with sufficient coverage of parental-specific reads. In the *Cr* × *Cg* endosperm, out of 158 sirenRNA loci (≥15 parental-specific reads), 103 clusters (65.19%) were maternally biased (maternal:paternal ratio, >4). Using the same criteria, in the *Cg* × *Cr* cross, out of 222 sirenRNA clusters, 184 clusters (82.88%) were maternally biased, and in the 4x*Cr* × *Cg* cross, out of 227 sirenRNAs, 152 clusters (66.96%) were maternally biased (Fig. 4f,g). Mapping reads from *Cr* × *Cr* and *Cg* × *Cg* to their respective genomes revealed variations in the level and specificity of sirenRNAs. Specifically, when *Cg* × *Cg* reads were mapped to the *Cr* genome, there was an apparent decrease in sirenRNA levels, which was also observed when mapping *Cr* × *Cr* reads to the *Cg* genome. These data reveal that at least some sirenRNAs are species-specific and that *Cg* accumulates substantially higher levels of sirenRNAs than *Cr* (Fig. 4b,e). This was also reflected in substantially higher siRNA levels in *Cg* × *Cr* hybrids at loci losing siRNAs in the reciprocal *Cr* × *Cg* hybrid (Extended Data Fig. 7a).

**Fig. 5:**
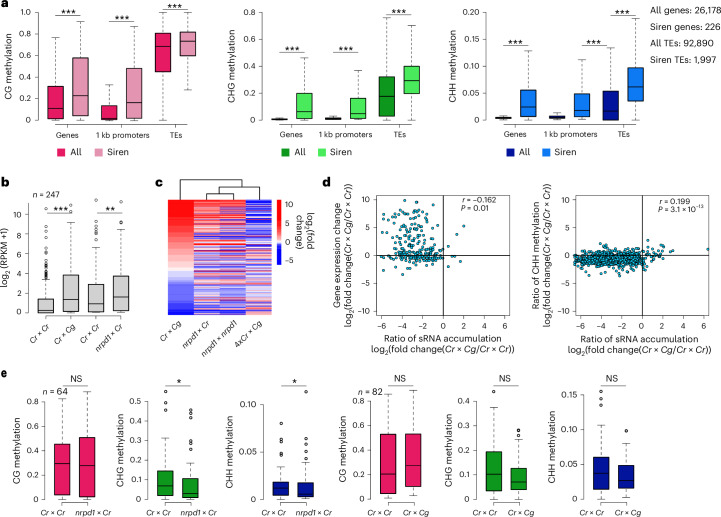
Loss of siRNAs correlates with changes in gene expression and DNA methylation. **a**, Comparison of DNA methylation levels in genes (gene body), promoters and TEs overlapping siren loci with all genes and TEs in the *Cr* genome. The asterisks indicate statistically significant differences as calculated by two-sided Wilcoxon tests (***P < 0.001). **b**, Expression of genes overlapping siren loci in *Cr* × *Cr*, *Cr* × *Cg* and *nrpd1* × *Cr* seeds at 6 DAP. The asterisks indicate statistically significant differences as calculated by two-sided Wilcoxon tests (***P < 0.001; **P < 0.01). **c**, Heat map and dendrogram show clustering of samples based on log_2_ fold changes (compared with the corresponding maternal parent) of genes overlapping siren loci. **d**, Scatter plots showing Pearson’s correlation (*r*) (two-sided) between the loss of siRNAs and changes in genes expression (left) and CHH methylation (right). **e**, Comparison of DNA methylation in *Cr* × *Cr* versus *nrpd1* × *Cr* (left) and *Cr* × *Cr* versus *Cr* × *Cg* (right) endosperm on upregulated genes (in the respective crosses) overlapping siren loci. The asterisks indicate statistically significant differences as calculated by a two-sided Wilcoxon test (*P < 0.05). In **a**, **b** and **e**, the horizontal lines show the median values, the box edges show the interquartile range and the whiskers show the full range excluding outliers. Source data

Siren loci were heavily methylated, corresponding to the production of high levels of sirenRNAs (Figs. 4e and 5a). The depletion of sirenRNAs in *Cr* × *Cg* and *nrpd1* × *Cr* endosperm corresponded to increased mRNA levels of many genes overlapping those loci (Fig. 5b–d), indicating that sirenRNAs negatively regulate gene expression, consistent with previous work^29,30^. Transcriptional changes were related to a loss of CHG and CHH methylation; however, this loss was not significant in the hybrid endosperm (Figs. 4b and 5e).

The depletion of maternal 24-nucleotide siRNAs in the endosperm of hybrid tomato seeds was related to decreased expression of Pol IV-subunit-encoding genes *NRPD1* and *NRPD2* (ref. ^31^). We tested the expression of RdDM components in hybrid seed transcriptomes and found significantly reduced *NRPD1* transcript levels in *Cr* × *Cg*, *Co* × *Cr* and *Cr* × 4x*Cr* endosperm (Extended Data Fig. 7c). However, we also found significantly reduced *NRPD1* transcripts in 4xCr × *Cr* endosperm (Extended Data Fig. 7c), making it unlikely that reduced *NRPD1* expression is causally responsible for the loss of sirenRNAs in the hybrids.

### Depletion of sirenRNAs causes loss of methylation at *trans* targets

**Fig. 6:**
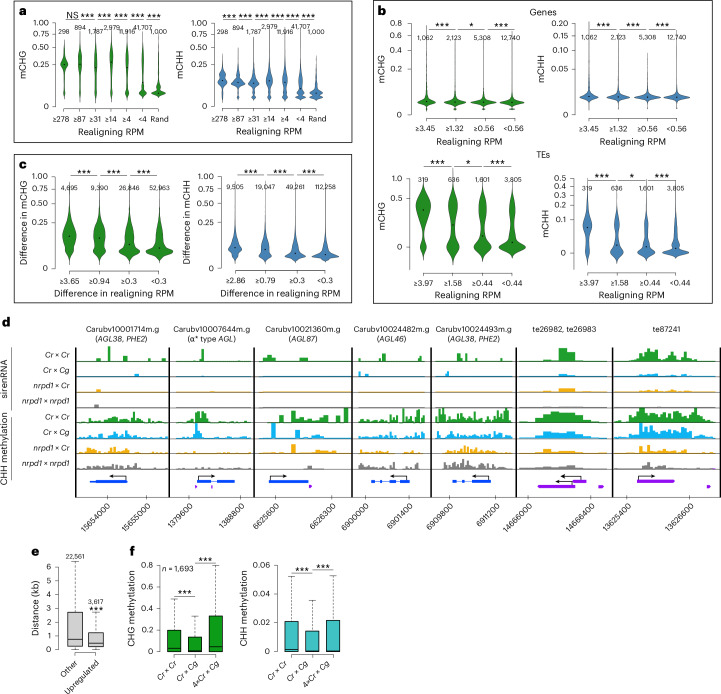
Loss of DNA methylation at *trans* sirenRNA targets. **a**, CHG and CHH DNA methylation at sirenRNA *trans* targets (50-bp genomic windows, with ≥1 RPM realigning sirenRNAs and ≤2 mismatches) in *Cr* × *Cr* endosperm. The numbers on the plots show the number of windows in each category; the breaking points correspond to 0.7, 0.9, 0.98 and 0.995 quantiles. Rand, random. **b**, CHG and CHH DNA methylation at sirenRNA *trans* targets overlapping genes (coding region +2 kb promoters; top) and TEs (bottom). The numbers on the plots show the number of windows in each category; the breaking points correspond to 0.5, 0.8 and 0.9 quantiles. **c**, Comparison of CHG and CHH DNA methylation levels in 50-bp windows differing in sirenRNA accumulation at *trans* targets. The *x* axis shows the difference in DNA methylation (*Cr* × *Cr* minus *Cr* × *Cg*), and the *y* axis shows the difference in sirenRNA accumulation in *trans* (*Cr* × *Cr* minus *Cr* × *Cg*). The numbers correspond to windows in each category; the breaking points correspond to 0.6, 0.85 and 0.95 quantiles. In **a**–**c**, the asterisks indicate statistically significant differences as calculated by two-sided Wilcoxon tests with Benjamini and Hochberg correction for multiple comparisons (***P < 0.001; *P < 0.05). **d**, Examples of genes and TEs with reduced sirenRNA accumulation at *trans* targets in *Cr* × *Cg*, *nrpd1* × *Cr* and *nrpd1* × *nrpd1* compared with *Cr* × *Cr* endosperm. The tracks show levels of sirenRNAs and CHH methylation at the indicated genes and TEs. The blue boxes represent genes; the purple boxes represent TEs. **e**, Distance to the nearest TE upstream of the transcriptional start site of upregulated genes in *Cr* × *Cg* compared with non-upregulated genes. The asterisks indicate statistically significant differences as calculated by a two-sided Wilcoxon test (***P < 0.001). **f**, Methylation levels of TEs in the promoters of upregulated genes in the *Cr* × *Cg* hybrid. Statistical differences were calculated with two-sided Wilcoxon’s tests for paired samples. In **e** and **f**, the horizontal lines show the median values, the box edges show the interquartile range and the whiskers show the full range excluding outliers. Source data

**Extended Data Fig. 8:**
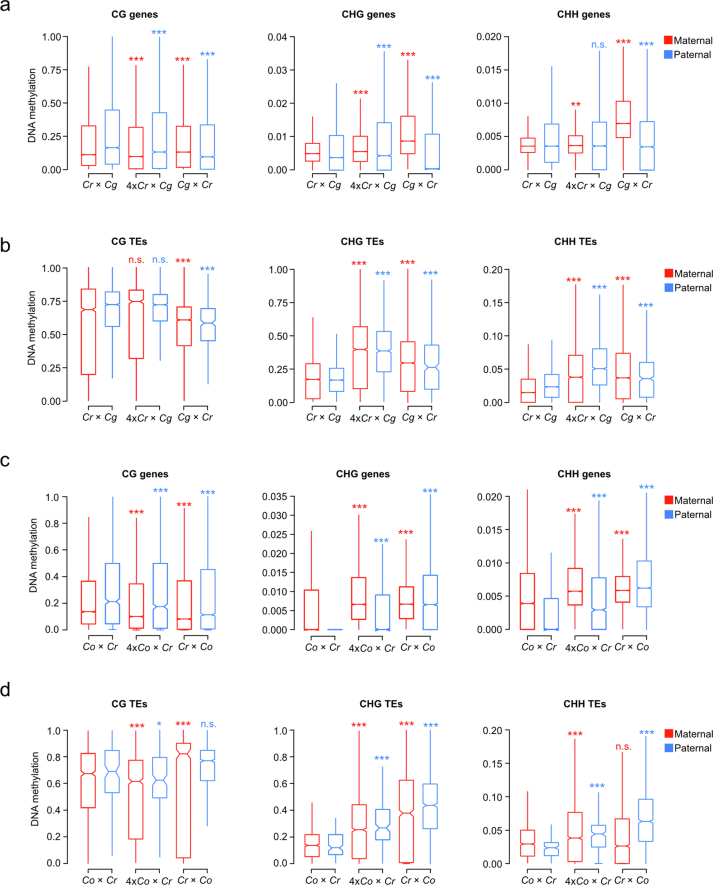
Parental-specific DNA methylation in the hybrid endosperm of *Capsella* crosses. **a**, **b**, Boxplot show parental-specific DNA methylation levels of genes (**a**) and TEs (**b**) in the endosperm of *Cr* × *Cg*, 4x*Cr* × *Cg* and *Cg* × *Cr* at 6 DAP. Maternal and paternal methylation values in each genotype are compared to the respective parent in the *Cr* × *Cg* cross. Asterisks indicate statistically significant differences as calculated by the two-sided Wilcoxon test, p-values were adjusted for multiple comparisons with Benjamini and Hochberg correction (p-value: ***< 0.001, **< 0.01, *< 0.05, n.s. – not significant). **c**, **d**. Boxplot show parental-specific DNA methylation levels of genes (**c**) and TEs (**d**) in the endosperm of *Co* × *Cr*, 4x*Co* × *Cr* and *Cr* × *Co* at 6 DAP. Maternal and paternal methylation values in each genotype are compared to the respective parent in *Co* × *Cr* cross. Asterisks indicate statistically significant differences as calculated by the two-sided Wilcoxon test, p-values were adjusted for multiple comparisons with Benjamini and Hochberg correction (p-value: ***< 0.001, **< 0.01, *< 0.05, n.s. – not significant). **a**-**d**, Boxes show median values and the interquartile range. Whiskers show the full range excluding outliers. Source data

Previous work revealed that sirenRNAs can methylate other genomic sequences in *trans*^29^. We identified sirenRNA *trans* targets by mapping sirenRNAs to the genome masked for sirenRNA-producing loci by allowing two mismatches. We found that loci accumulating higher levels (≥4) of non-perfectly matching sirenRNAs had higher methylation levels in the CHG and CHH contexts (Fig. 6a). SirenRNAs targeted genes and TEs, with dosage-dependent effects on DNA methylation being most prominent on TEs (Fig. 6b). Together, these data strongly suggest that sirenRNAs guide DNA methylation in *trans* in the endosperm, like their proposed function in ovules^29^. Consistent with this idea, we found a decline of CHG and CHH methylation at *trans* targets in *Cr* × *Cg* hybrid endosperm that correlated with the dosage of depleted sirenRNAs (Fig. 6c,d). Conversely, the gain of CHG and CHH methylation upon increased maternal ploidy (4x*Cr* × *Cg* and 4x*Co* × *Cr*) and in maternal-excess hybrids (*Cg* × *Cr* and *Cr* × *Co*) occurred on both maternal and paternal alleles (Extended Data Fig. 8), consistent with the idea that maternal siRNAs have *trans*-acting activity.

We next asked whether there is a connection between sirenRNAs, loss of DNA methylation at *trans* targets and changes in gene expression in the hybrid *Cr* × *Cg* endosperm. Consistent with sirenRNAs most prominently targeting TEs (Fig. 6b), we found that genes upregulated in *Cr* × *Cg* hybrids were closer to a TE upstream of their transcription start site than other genes (Fig. 6e). Moreover, the TEs in the promoters of those genes had reduced CHG and CHH methylation in *Cr* × *Cg* hybrids (Fig. 6f). Among the genes that were targeted by *trans*-acting sirenRNAs and lost DNA methylation we found many upregulated *AGL*s (Extended Data Fig. 4a,b and Supplementary Data 3), consistent with previous findings reporting targeting of *AGL*s by maternal Pol IV-dependent siRNAs^30^.

**Extended Data Fig. 9:**
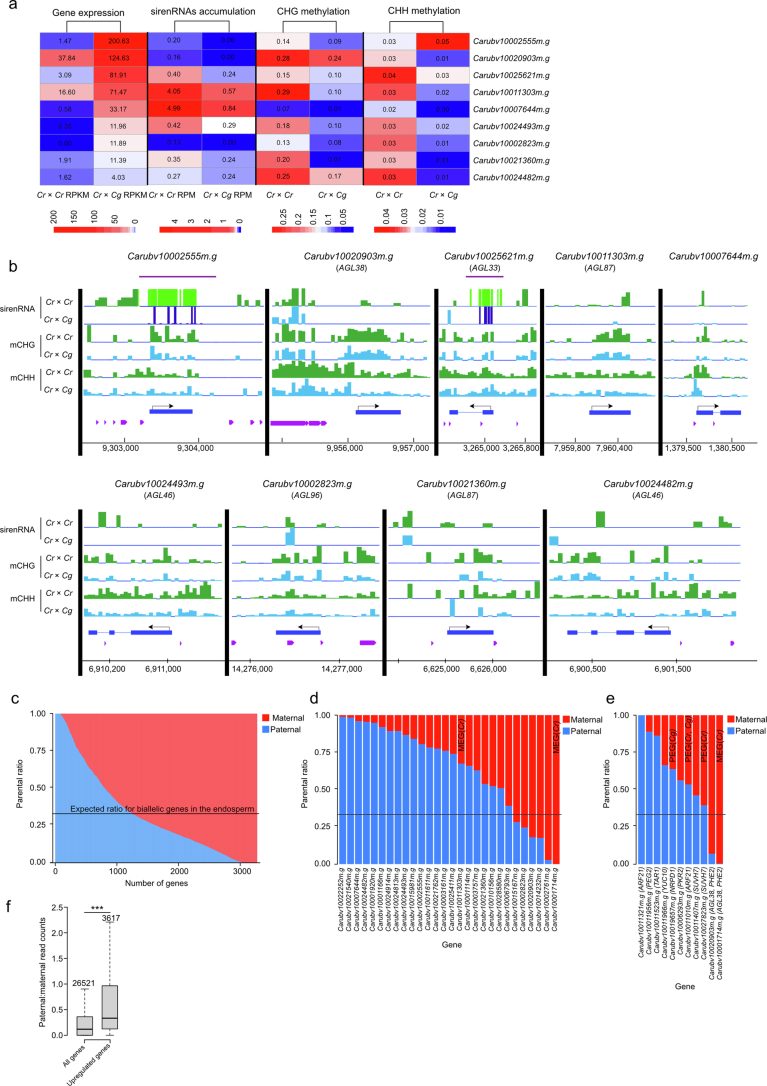
Characterization of sirenRNAs trans-targets and parental-specific analysis of gene expression in *Cr* × *Cg.* **a**, Heatmaps show expression of *AGL* genes (RPKM) targeted by *trans*-acting sirenRNAs, accumulation of sirenRNAs at *trans AGL* targets and corresponding CHG and CHH methylation. **b**, Tracks show sirenRNA accumulation at *trans AGL* targets and corresponding CHH and CHG methylation levels (same set of genes as on panel **a**). The purple line above the tracks marks *cis* acting siren RNAs. Light green and navy-blue bars on sirenRNA tracks show reads mapping in *cis* at siren loci. **c**-**e**, Barplots show the proportion of maternal and paternal reads in genes upregulated in *Cr* × *Cg* (**c**), γ-type *AGLs* upregulated in *Cr* × *Cg* (**d**) and selected orthologs of genes that have been previously reported as imprinted and involved in endosperm development or the triploid block in Arabidopsis (**e**). Expression data are obtained from whole seeds at 6 DAP. Black horizontal line on each plot represents the expected 2:1 ratio (maternal:paternal) for biallelic genes in the endosperm. **f**, Boxplot show the ratio of paternal:maternal read counts for all genes and genes upregulated in *Cr* × *Cg*. Boxes show median values and the interquartile range. Whiskers show the full range excluding outliers. Asterisks indicate statistically significant differences as calculated by the two-sided Wilcoxon test (*** p-value < 0.001). Source data

On the basis of these data, we propose that sirenRNAs in the *Capsella* endosperm guide non-CG DNA methylation in *trans*. Depletion of sirenRNAs in the *Cr* × *Cg* hybrid leads to loss of DNA methylation and increased expression of several *trans* targets, among them several genes with potential function in establishing hybridization barriers (Supplementary Data 3). These include *SUVH7*, which is involved in the triploid block in *Arabidopsis*^32^, *YUC10*, an auxin biosynthesis gene^33^ and *ARF21*—a centromeric ARF regulating endosperm cellularization in *Arabidopsis*^34^. Moreover, among those genes are nine *AGL*s including *AGL38*—the orthologue of *PHE1* (Extended Data Fig. 9a,b), which activates key regulators of the triploid block in *Arabidopsis*^35^. Most of these genes had paternally biased expression in the hybrid endosperm, consistent with the paternal-excess phenotype (Extended Data Fig. 9c–f).

### Putative AGL targets are upregulated in hybrid endosperm

**Fig. 7:**
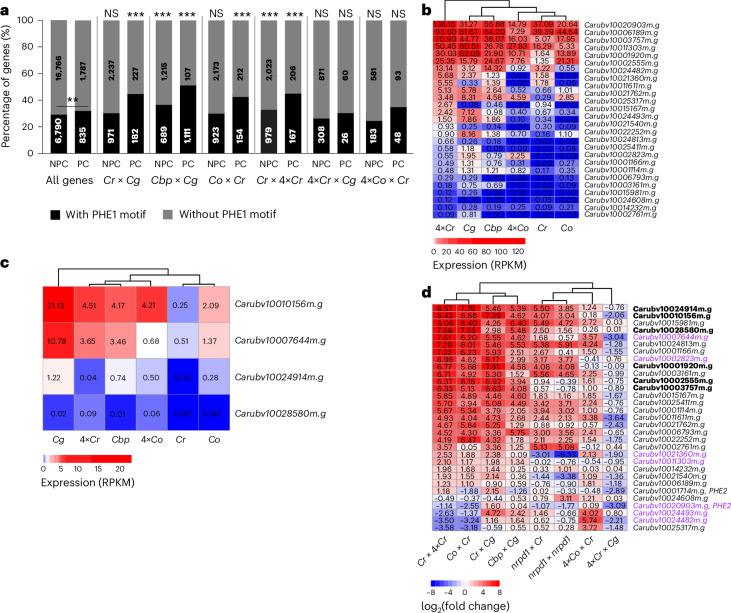
Increased expression of *AGL*s correlates with increased expression of AGL targets in pericentromeric regions. **a**, Distribution of PHE1 binding motifs in the *Capsella* genome in different groups of genes: all genes and genes upregulated in *Cr* × *Cg*, *Cbp* × *Cg*, *Co* × *Cr*, *Cr* × 4x*Cr*, 4x*Cr* × *Cg* and 4x*Co* × *Cr*. The numbers of pericentromeric (PC) and non-pericentromeric (NPC) genes upregulated in different genotypes are compared with the numbers of all pericentromeric/non-pericentromeric genes. The asterisks indicate statistically significant differences calculated by two-sided chi-square tests with Benjamini and Hochberg correction for multiple comparisons (***P < 0.001). The numbers show the number of genes in each category. **b**,**c**, Heat maps and dendrograms show clustering of genes based on the expression (RPKM) of type I γ-MADS-box (**b**) and α*-MADS-box (**c**) transcription factors in different *Capsella* genotypes. **d**, Heat map and dendrogram show clustering of genes based on log_2_(fold change) (in comparison to the maternal parent) of γ and α* type I MADS-box transcription factors in different *Capsella* hybrids. Genes marked in bold overlap siren loci; genes marked in purple are targeted by *trans*-acting sirenRNAs. Source data

Previous work revealed that target sequence motifs of the AGL PHE1 are frequently located in helitron TEs^35^. Since helitrons in *Arabidopsis* and other Brassicaceae are concentrated in pericentromeric regions^36,37^, we tested whether pericentromeric regions in *Capsella* were enriched for PHE1 binding motifs. We found that the frequency of genes containing PHE1 binding motifs was significantly higher in pericentromeric regions than in chromosome arms (Fig. 6g). Importantly, in all analysed incompatible crosses, the promoter regions of upregulated genes located in pericentromeric regions were significantly enriched for PHE1 binding sites (Fig. 6g). On the basis of these data, we propose that the increased expression of genes in pericentromeric regions in hybrid endosperm is a consequence of the increased expression of *AGL*s and their ability to target pericentromeric regions due to the loss of DNA methylation^9^. DNA methylation was shown to antagonize the binding of PHE1 (ref. ^35^), supporting the proposed antagonism of DNA methylation and AGL binding. Our data (Extended Data Fig. 9a,b) and previously published data show that sirenRNAs negatively regulate *AGL*s in the endosperm^30^. Nevertheless, while the loss of maternal Pol IV function delays endosperm cellularization (Extended Data Fig. 5d), it does not cause complete endosperm cellularization failure^38^, suggesting an additional component causing this phenotype in paternal-excess hybrids. It has been shown that many *AGL*s have higher expression levels in *Cg* than in *Cr*^6^. Similarly, many PHE1-like (Mγ-type) *AGL*s are more highly expressed in 4x*Cr* than in 2x plants, resembling the profile of *Cg AGL* genes (Fig. 6h). The same pattern applied for Mα*-like *AGL*s (Fig. 7c), which encode potential heterodimerization partners of Mγ-type AGLs^10^. On the basis of these data, we propose that the increased expression of type I *AGL* genes in *Cg* and 4x*Cr* causes increased expression of *AGL* targets in hypomethylated regions (Fig. 7a). The allopolyploid *Cbp* behaved like *Cr* in crosses with *Cg* (Fig. 1a,c), which was reflected by reduced expression of several *AGL*s in *Cbp* compared with *Cg*. These data support the idea that increased dosage of *AGL*s in hybrid endosperm is a critical determinant of hybrid seed arrest. Similarly, the expression of Mγ- and Mα*-type *AGL*s in *Co* was lower than in *Cg*, resembling that of *Cr*. While several *AGL*s were more highly expressed in *Co* than in *Cr*, the *CrPHE1* orthologue Carubv10020903m.g^9^ was nearly twice as strongly expressed in *Cr* (Fig. 7b), possibly connecting to the paternal-excess effect of *Cr* when crossed to *Co* (Fig. 1a). Furthermore, the majority of *AGL*s were upregulated to substantially higher levels in paternal-excess hybrid seeds than in *nrpd1* seeds, supporting the idea that the increased expression of *AGL*s determines the phenotypic difference between paternal-excess hybrid seeds and seeds lacking maternal NRPD1 function (Fig. 7d). Importantly, increased maternal genome dosage suppressed *AGL* expression in 4x*Cr* × *Cg* and 4x*Co* × *Cr* hybrids (Fig. 7d), which was connected with increased DNA methylation in the promoter regions of *AGL* genes (Extended Data Fig. 7b) and restored seed viability^21^ (Fig. 1d).

Together, on the basis of our data, we propose that seed abortion in response to paternal-excess interspecies and interploidy hybridizations is triggered by two factors: the depletion of maternally derived sirenRNAs and the increased expression of *AGL*s targeting hypomethylated regions in hybrid endosperm.

## Discussion

In this study, we uncover a connection between the dosage of maternal Pol IV-dependent sirenRNAs and hybridization success in *Capsella*. Our findings demonstrate that depletion of sirenRNAs connects to chromatin decondensation and the loss of non-CG DNA methylation in the endosperm, resembling phenotypes of paternal-excess hybrid endosperm. Consistent with the similar molecular and chromatin phenotypes observed in hybrid paternal-excess and maternal *nrpd1* mutant endosperm, there was a strong depletion of sirenRNAs in the hybrid, underscoring the impact of hybridization on sirenRNA production. This depletion of sirenRNAs in both hybrid and maternal *nrpd1* mutant endosperm correlated with a substantial increase in mRNA levels of genes overlapping siren loci, highlighting the potential role of sirenRNAs as negative regulators of gene expression. Siren loci exhibit extensive methylation in wild-type endosperm (Figs. 4d,e and 5a). The transcriptional alterations observed in *nrpd1* and hybrid endosperm align with a reduction of DNA methylation at siren loci, providing strong support for the role of sirenRNAs in directing DNA methylation. Our data also suggest, in line with previous findings in male meiocytes and ovules^29,39^, that sirenRNAs may act in *trans* by guiding DNA methylation to non-perfectly matching TEs and protein-coding genes. Genes targeted by sirenRNAs that undergo upregulation in hybrid endosperm encompass several genes with potential involvement in establishing hybridization barriers, including *SUVH7*, *YUC10*, *ARF21* and *PHE1* orthologues^32,34,35^. This strongly implies a direct connection between the depletion of sirenRNAs and the arrest of hybrid seed development.

SirenRNAs, originating predominantly from the maternal source (ref. ^16^ and our own data), probably guide methylation in the endosperm post-fertilization. Since heightened maternal genome dosage can ameliorate hybrid seed defects while concurrently inducing DNA hypermethylation (Fig. 2d,e and Extended Data Fig. 3), we posit that dosage of maternally provided sirenRNAs is insufficient to repress sirenRNA targets in paternal-excess hybrids. Consistently, interploidy *Cr* × 4x*Cr* seeds exhibit similar molecular aberrations to *Cr* × *Cg* hybrids, strongly corroborating the notion that the relative dosage of maternal sirenRNAs to their targets is a key determinant of hybridization success. The loss of RDR2, which together with Pol IV is required for siRNA production^13,40^, causes strong seed defects in *Cg*, differing from the mild defects of *rdr2* mutants in *Cr*^41^. These data align with our finding that *Cg* produces higher levels of sirenRNAs than *Cr*, suggesting that sirenRNA levels adapt to the expression strength of their targets. Among those targets are *AGL* genes that we found to scale in expression with the effective ploidy of *Capsella* species (*Co* < *Cr* = *Cbp* < *Cg* = 4x*Cr*). The differential expression levels of *AGL*s in *Capsella* are probably driven by mating system divergence. According to theoretical predictions, genes promoting endosperm growth are more strongly expressed in outbreeding than in inbreeding species^22^. Consistent with these predictions, *Cg* is an outbreeder and has the strongest *AGL* expression levels, followed by the recent inbreeders *Cr* and *Cbp* and finally by the ancient inbreeder *Co* (Fig. 7b).

The maternal *nrpd1* mutant exhibited a phenotype mirroring that of paternal-excess hybrids in terms of alterations in DNA methylation and chromatin condensation. The seeds of *nrpd1* mutants cellularize later than wild-type seeds and also abort, albeit at a lower frequency than interspecies hybrids (Extended Data Fig. 5). One probably decisive difference between hybrid and *nrpd1* endosperm is the extent of *AGL* deregulation, which may account for the difference in seed abortion frequency. While *AGL*s targeted by sirenRNAs were also upregulated in *nrpd1* endosperm, most experienced higher upregulation in the hybrid endosperm (Fig. 7d). This discrepancy is probably attributed to the amplified *AGL* expression in the paternal parents of hybrids, inducing a more pronounced response in hybrid endosperm than in the *nrpd1* mutant.

Given that AGL target genes are notably concentrated in pericentromeric regions, the combined effects of diminished DNA methylation and increased AGL activity probably account for the heightened expression of AGL targets in paternal-excess hybrids. The same scenario applies to interploidy *Cr* × 4x*Cr* seeds, which exhibit a substantial overlap in hyperactivated AGL target genes with paternal-excess hybrids. Mutations in several *AGL*s were shown to weaken barriers associated with interploidy and interspecies hybridization^35,42,43^, providing further support for this scenario. Since Mγ AGLs have strongly expanded in *Capsella* and most of them are highly upregulated in the hybrids (Fig. 7d), directly testing the functional relevance of AGLs in establishing interspecies hybridization barriers in *Capsella* is challenging, but this remains an important task of future studies. Taken together, on the basis of our findings, we propose that maternal sirenRNAs serve as a dosage-sensitive factor that decisively influences the success of hybridization between plant species.

## Methods

### Plant material and growth conditions

The following accessions of different *Capsella* species have been used in this study: *Cr* 48.21, 4x*Cr* 48.21, *Cbp* 27.4, *Cg* 23.5 and *Co* 1719. Tetraploid *Co* seeds were provided by M. Lascoux^44^. The seeds were surface sterilized with 30% bleach and 70% ethanol, rinsed with distilled water and sown on agar plates containing ½ Murashige and Skoog medium and 1% sucrose. The seeds were then stratified for two days in the darkness at 4 °C and moved into a growth chamber with a long-day photoperiod (16 h and 22 °C light, 8 h and 19 °C darkness) with a light intensity of 110 µE. Seven-day-old seedlings were transferred to pots filled with sterile soil, and the plants were grown in a growth chamber with 60% humidity and daily cycles of 16 h light at 21 °C and 8 h darkness at 18 °C with a light intensity of 150 µE. For germination tests, dry seeds were stored for 30 days to break dormancy. The seeds were then surface sterilized and sown on agar plates as described above. The seeds were stratified for two days at 4 °C and then moved to a growth chamber and scored after seven days for seedling establishment.

### Histological and fluorescence analyses

Manually pollinated siliques were harvested after 4–7 DAP and processed for clearing and Feulgen staining as previously described^33^. The siliques were opened at the side with needles and incubated overnight in fixing solution (ethanol:acetic acid (3:1)) at 4 °C. The samples were washed with distilled water three times for 15 minutes and then incubated for 1 h in freshly prepared 5 N HCl. After incubation, the samples were washed again with distilled water three times for 15 minutes and incubated with Schiff reagent (Sigma-Aldrich) for 3–4 h. The samples were washed with cold (4 °C) distilled water three times for 10 minutes and then washed in different concentrations of ethanol (10%, 30%, 50%, 70% and 95%) for 10 minutes in each solution. The samples were then washed with 99.5% ethanol until they remained colourless (at this point samples can be stored in this solution overnight at 4 °C). The samples were incubated in 99.5% ethanol:LR White (London Resin White + catalyst, Sigma Aldrich) 3:1 and 2:1, 15 minutes in each solution. The samples were incubated in 1:1 ethanol:LR White for 1 h and then in LR White overnight. Seeds were taken out of the siliques, mounted on a microscope slide in a drop of LR White + catalyst and baked at 60 °C for 16 h for polymerization. The slides were watched under a two-photon microscope with an excitation wavelength of 800 nm and emission from 518 nm and onwards.

### *Capsella grandiflora* sequencing and genome assembly

DNA was extracted from young leaf material of *Cg* using the CTAB method^45^. DNA was sized on the BluePippin system (Sage Science) to remove small fragments and then sequenced on one Oxford Nanopore PromethION flow cell. The pore version used was R9.4.1, and the PromethION release version was 19.05.1. A total of 49.2 Gb was sequenced, corresponding to about 200× coverage of the estimated genome size of approximately 250 Mb (roughly calculated by flow cytometry analysis). Additionally, *Cg* DNA was used to prepare an Illumina overlap library. DNA was sheared using a Covaris M220 (Covaris) to 450 bp. The sheared DNA was sized using a BluePippin prep system and used to prepare a PCR-free Illumina sequencing library. The library was sequenced on the Illumina NovaSeq SP platform using an SP flow cell and 2 ×250 bp protocol.

A selection of the longest Oxford Nanopore PromethION sequence reads, together representing 60× haploid genome coverage, were assembled using Minimap2 (v.2.16-r922, with settings *m*, 1,600; K2G; I8G)^46^ and Miniasm (v.0.2-r137-dirty, with settings R; *c*, 2; *m*, 500; *s*, 4,000)^47^. A consensus sequence was generated through three iterations of Racon (v.1.4.10, with the default settings)^48^, using all sequence reads. The Illumina read pairs were then used to polish the consensus through three rounds of BWA-mem (v.0.7.17, with the default parameters)^49^ and Pilon (v.1.22, with the default parameters)^50^. Finally, purge_dups^51^ was used (v.15082019, with the default parameters) to generate a haploid representation of the heterozygous *Cg* assembly.

### RNA-seq

Seeds derived after manual pollination were dissected out of siliques at 6 DAP and collected in 20 µl of RNAlater solution (Sigma-Aldrich). Each sample consisted of 10–15 siliques. RNA was extracted using the RNeasy Plant Mini Kit (Qiagen) according to the manufacturer’s instructions. 500 ng of RNA was used for RNA-seq library preparation using the NEBNext Poly(A)mRNA Magnetic Isolation Module and NEBNext Ultra RNA LibraryPrep Kit for Illumina. Three biological replicates were generated for each genotype. The libraries were sequenced on an Illumina HiSeq X machine in 150-bp paired-end mode.

### RNA-seq analysis

Adapter trimming was performed using Trim galore with the following parameters: three_prime_clip_R1, 15; three_prime_clip_R2, 15; clip_R1, 10; clip_R2, 10. Sequencing reads were aligned to the *Cr* genome v.1.0 (Phytozome) using HISAT2 (ref. ^52^). Reads were assigned to genes with featureCounts from the Bioconductor Rsubread package^53^. Differentially regulated genes were detected using DESEQ2 (ref. ^54^). Genes were considered as upregulated on the basis of log_2_(fold change) > 1 and *P*_adj_ < 0.05 in comparison to the maternal parent (for incompatible hybrids) or to *Cr* × *Cr* (for crosses with the *nrpd1* mutant). Pericentromeric regions were defined as in ref. ^55^.

### Bisulfite sequencing

For bisulfite sequencing (BS-seq), seeds derived after manual pollination were dissected out of siliques at 6 DAP, and the endosperm was manually dissected as previously described^25^. Manually pollinated seeds 6 DAP were removed from siliques and put on a microscopic slide covered with a piece of tissue paper soaked with a drop of RNAlater solution (Sigma-Aldrich). The seeds were gently squashed with another microscopic slide to release the endosperm, and the embryos and seed coats were removed with tweezers. Tissue paper with the endosperm was put in an Eppendorf tube and frozen in liquid nitrogen. Approximately 600 seeds were used per replicate. The samples were stored at −70 °C and then used for genomic DNA isolation using the DNeasy Plant Mini Kit (Qiagen). Biological duplicates were generated for each genotype. Libraries were prepared with the Accel-NGS Methyl-Seq DNA Library Kit from Illumina, and the sequencing was performed on an Illumina NovaSeq 6000 machine in 150-bp paired-end mode.

### Bioinformatic analysis of BS-seq data

For DNA methylation analysis, the 150-bp-long paired-end reads were first quality trimmed by removing the first 5 bases from the 5′ end and the last 15 bases from the 3′ end. Reads were mapped to the *Cr* reference genome in paired-end mode (score_min L, 0–0.6) using Bismark v.0.16.3 (ref. ^56^). The mapped reads were deduplicated, and cytosine methylation values calculated using the Bismark Methylation Extractor.

Differentially methylated regions in the CG, CHG and CHH contexts were calculated using 50-bp windows across the genome as units. Only hypomethylated regions (*Cr* × *Cr* > *Cr* × *Cg*, *Cr* × *Cr* > *Cr* × 4x*Cr* and *Cr* × *Cr* > *nrpd1* × *Cr*) were considered. Windows with differences in fractional methylation below the first decile (Fisher’s exact test *P* < 0.01) were selected, and these were merged if they occurred within 300 bp.

### Parental-specific analysis of RNA-seq and BS-seq data

Parental gene expression and parental methylation analyses in plant crosses were performed using the allele-specific alignment sorter SNPsplit^57^. A masked *Cr* genome with SNP positions masked by the ambiguity base ‘N’ were constructed to run SNPsplit in the crosses with *Cg* and *Co*. To define SNPs, we performed an ordinary Illumina resequencing of *Cg* and *Co* plants. Reads from both species were quality trimmed with trimgalore (clip_R1, 15; clip_R2, 15; three_prime_clip_R1, 5; three_prime_clip_R2, 5) and mapped to the *Cr* reference genome using BWA^49^ in PE mode. SNP calling was performed using freebayes^58^(iXu; *G*, 20; *F*, 0.9), and masked genomes were built using bedtools maskfasta^59^. The RNA-seq analysis used hisat2 (ref. ^52^), and the BS-seq analysis used bismark (v.0.16.3)^56^ as aligners, before the SNPsplit parental read sorting. Gene expression was quantified with htseq-count^60^.

### Small RNA sequencing

Seeds derived after manual pollination were dissected out of siliques at 6 DAP and squeezed on facial tissue paper to release the endosperm. Seed coats and embryos were removed with forceps. A drop of RNAlater solution (Sigma-Aldrich) was added to the tissue containing the absorbed endosperm, transferred to an Eppendorf tube and frozen in liquid nitrogen. Each sample consisted of endosperm from about 600 seeds. Small RNAs were extracted using the mirVana miRNA Isolation Kit (ThermoFisher Scientific) following the manufacturer’s instructions for sRNA. Libraries were prepared using the NEBNext Multiplex Small RNA Library Prep Set for Illumina with 50–100 ng of input for each sample. Size selection was performed on a 6% polyacrylamide gel, and bands corresponding to about 150 bp were cut and purified from the gel for further analysis. Sequencing was performed on a NovaSeq 6000 machine in 150-bp paired-end mode.

### Bioinformatic analyses of sRNA-seq data

Adapters were removed from the first read of the 150-bp long read pair of each library using cutadapt, and the resulting 18–25-bp long reads were selected. Reads belonging to chloroplasts, mitochondria and structural non-coding RNAs (tRNAs, snRNAs, rRNAs or snoRNAs) were removed using bowtie (v.1).

The remaining reads were mapped to the *Cr* or *Cg* genome (see above), and sRNA loci were annotated with ShortStack^27,61^ using filtered reads from all generated libraries and sRNA from leaves^38^. The options used for ShortStack were mismatches, 0; mmap, u; mincov, 0.5 rpm; and pad, 75. Replicates were checked for consistency by principal component analysis using the vegan v.2.6-4 package in R^62^. Clusters with differential accumulation of sRNAs were identified with DESEQ2 (ref. ^54^). Genes and TEs were considered as depleted of sRNAs on the basis of log_2_(fold change) < 0 and *P*_adj_ < 0.1 in comparison to the maternal parent (for incompatible hybrids) or to *Cr* × *Cr* (for crosses with the *nrpd1* mutant).

### Parental-specific analysis of sRNA-seq data

Parental-specific analysis of sRNA was performed by mapping the 18–25-nucleotide sRNA population to the genomes of both parents with no mismatches (bowtie v.0)^63^ and selecting the reads that mapped exclusively to one parent and not to the other and vice versa. As parental genomes for *Co* and *Cg*, we used own-built ‘pseudogenomes’ in which the above-described SNPs between these species and *Cr* were substituted into the *Cr* reference genome.

### Endosperm nuclei spreading

Endosperm nuclei spreading was performed as previously described^9^. Manually pollinated seeds were harvested 4 DAP and incubated overnight in a solution containing 2.5 mM 8-hydroxyquinoline, 100 μM oryzalin and 100 μM colchicine. The seeds were subsequently fixed in ethanol:acetic acid (3:1) for at least five hours at 4 °C. Following fixation, the seeds were washed with 10 mM citrate buffer and incubated for five hours in an enzyme solution comprising 0.3% cytohelicase, 0.3% pectolyase and 0.3% cellulase in 10 mM citrate buffer. After enzymatic digestion, five to ten seeds were placed on a slide, squashed with a needle, spread using 60% acetic acid and fixed on the slide with ethanol:acetic acid (3:1). The slides were mounted using Vectashield mounting medium containing DAPI (BioNordika AB). The experiment was conducted with three independent biological replicates.

### Quantification of chromocenter condensation

Each endosperm nucleus was saved as a single .tif file and then analysed in Fiji^64^. Particle Analysis was run after threshold adjustment with MaxEntropy. Only particles larger than 0.05 µm^2^ were considered. The circularity of the particles was used as a proxy for chromocenter condensation as previously described^65–67^ with values ranging between 0 and 1 (1 corresponds to a perfect circle). Each nucleus was represented by the mean circularity of all chromocenters. For each genotype, around 50 nuclei were analysed.

### Motif search

PHE1 binding motifs were previously identified in ref. ^35^. The motif file was used for scanning promoter sequences (1 kb upstream of the ATG) of *Capsella* genes with the findMotifsGenome.pl function from HOMER^68^.

### Reporting summary

Further information on research design is available in the Nature Portfolio Reporting Summary linked to this article.

## Supplementary information

Supplementary Information Supplementary Figs. 1 and 2 and Table 1.

Reporting Summary

Supplementary Data 1 Gene expression in all analysed crosses. The table shows log_2_ fold changes of gene expression in the endosperm of the indicated crosses.

Supplementary Data 2 List of siren loci and overlapping genomic features. The table shows the positions and normalized read counts over siren loci in the endosperm of the indicated genotypes.

Supplementary Data 3 List of potential *trans* targets affected in the *Cr* × *Cg* hybrid. The table shows differences in sirenRNA accumulation (Diff_RPM > 0), differences in CHH and CHG methylation (diff_CHH > 0.01 OR diff_CHG > 0.05), and differences in RNA accumulation (log_2_ fold change > 1, p < 0.05) in *Cr* × *Cg* compared with *Cr* × *Cr*.

## Source data

Source Data Fig. 1 Statistical source data.

Source Data Fig. 2 Statistical source data.

Source Data Fig. 3 Statistical source data.

Source Data Fig. 4 Statistical source data.

Source Data Fig. 5 Statistical source data.

Source Data Fig. 6 Statistical source data.

Source Data Fig. 7 Statistical source data.

Source Data Extended Data Fig. 1 Statistical source data.

Source Data Extended Data Fig. 2 Statistical source data.

Source Data Extended Data Fig. 3 Statistical source data.

Source Data Extended Data Fig. 4 Statistical source data.

Source Data Extended Data Fig. 5 Statistical source data.

Source Data Extended Data Fig. 6 Statistical source data.

Source Data Extended Data Fig. 7 Statistical source data.

Source Data Extended Data Fig. 8 Statistical source data.

Source Data Extended Data Fig. 9 Statistical source data.

## Data Availability

The original data files for RNA-seq and DNA BS-seq can be obtained from the NCBI Gene Expression Omnibus (GSE246468). The assembled *Cg* genome sequence has been deposited at DDBJ/ENA/GenBank under the accession number JAVXYZ000000000. Source data are provided with this paper. The source data for Fig. 6a,c are also available via figshare at 10.6084/m9.figshare.27143508 (ref. ^69^).
