# Supplementary material for: Dosage-sensitive maternal siRNAs determine hybridization success in Capsella
Source: Nat Plants. 2024 Nov 11;10(12):1969–83. doi: 10.1038/s41477-024-01844-3 (PMC11649575; doi:10.1038/s41477-024-01844-3)
Supplement: Supplementary file 1 — Supplementary Figs. 1 and 2 and Table 1. [file 41477_2024_1844_MOESM1_ESM.pdf]

nature plants

Supplementary information https://doi.org/10.1038/s41477-024-01844-3

Dosage-sensitive maternal siRNAs determine hybridization success in *Capsella*

In the format provided by the authors and unedited

Supplementary Materials for

Dosage sensitive maternal siRNAs determine hybridization success in *Capsella*

K. Dziasek, J. Santos-González, K. Wang, Y. Qiu, J. Zhu, D. Rigola, K. Nijbroek, C. Köhler

Corresponding author: koehler@mpimp-golm.mpg.de

The PDF file includes:

Supplementary Fig 1 and 2

Supplementary Table 1

Other Supplementary Materials for this manuscript include the following:

Supplementary Data 1-3

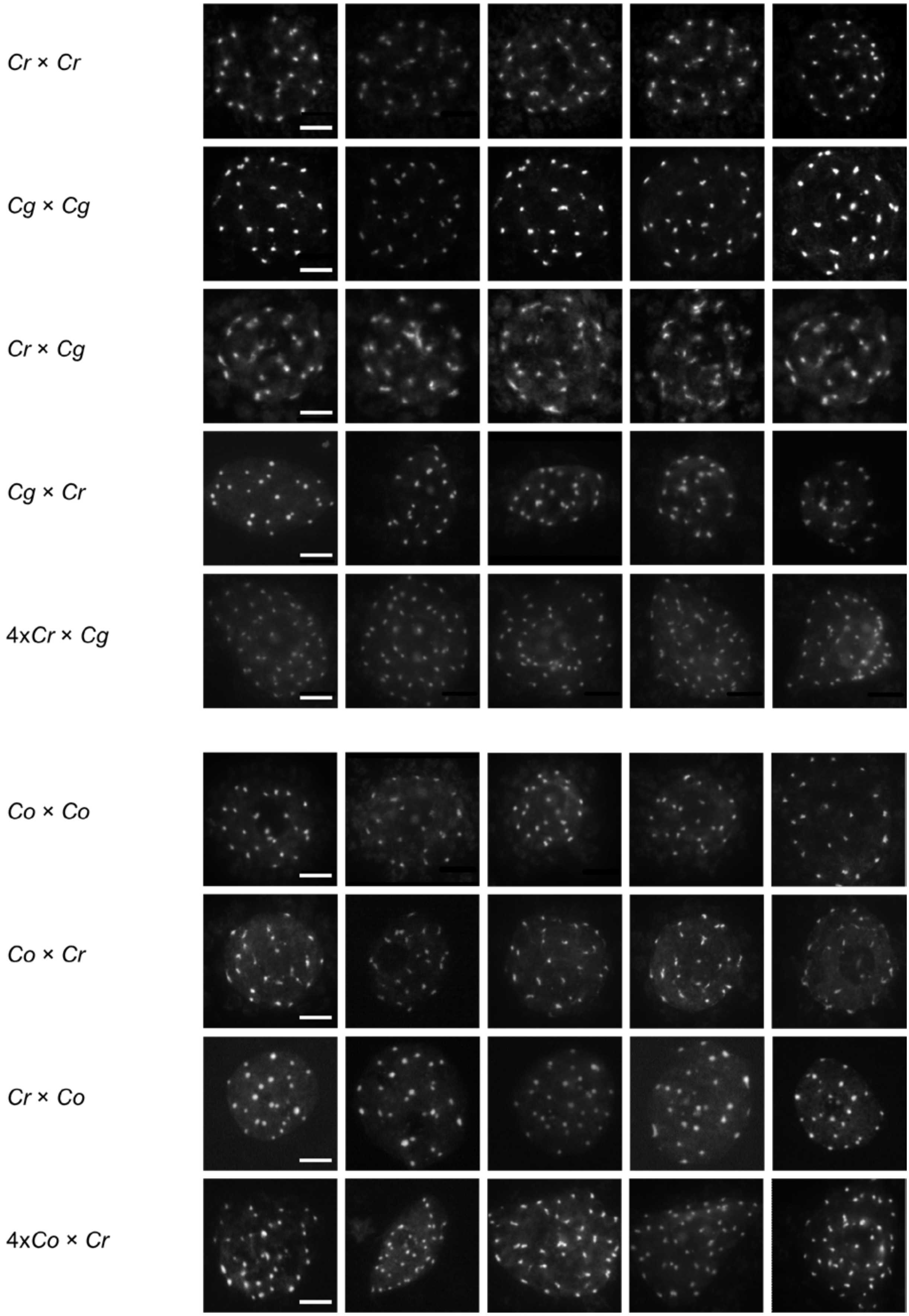
Cr × Cr
Cg × Cg
Cr × Cg
Cg × Cr
4xCr × Cg
Co × Co
Co × Cr
Cr × Co
4xCo × Cr

Supplementary Fig. 1 Chromatin condensation of endosperm nuclei in different *Capsella* genotypes.

Representative pictures of DAPI stained chromocenters of endosperm nuclei from different *Capsella* genotypes. Scale bar, 5 μm.

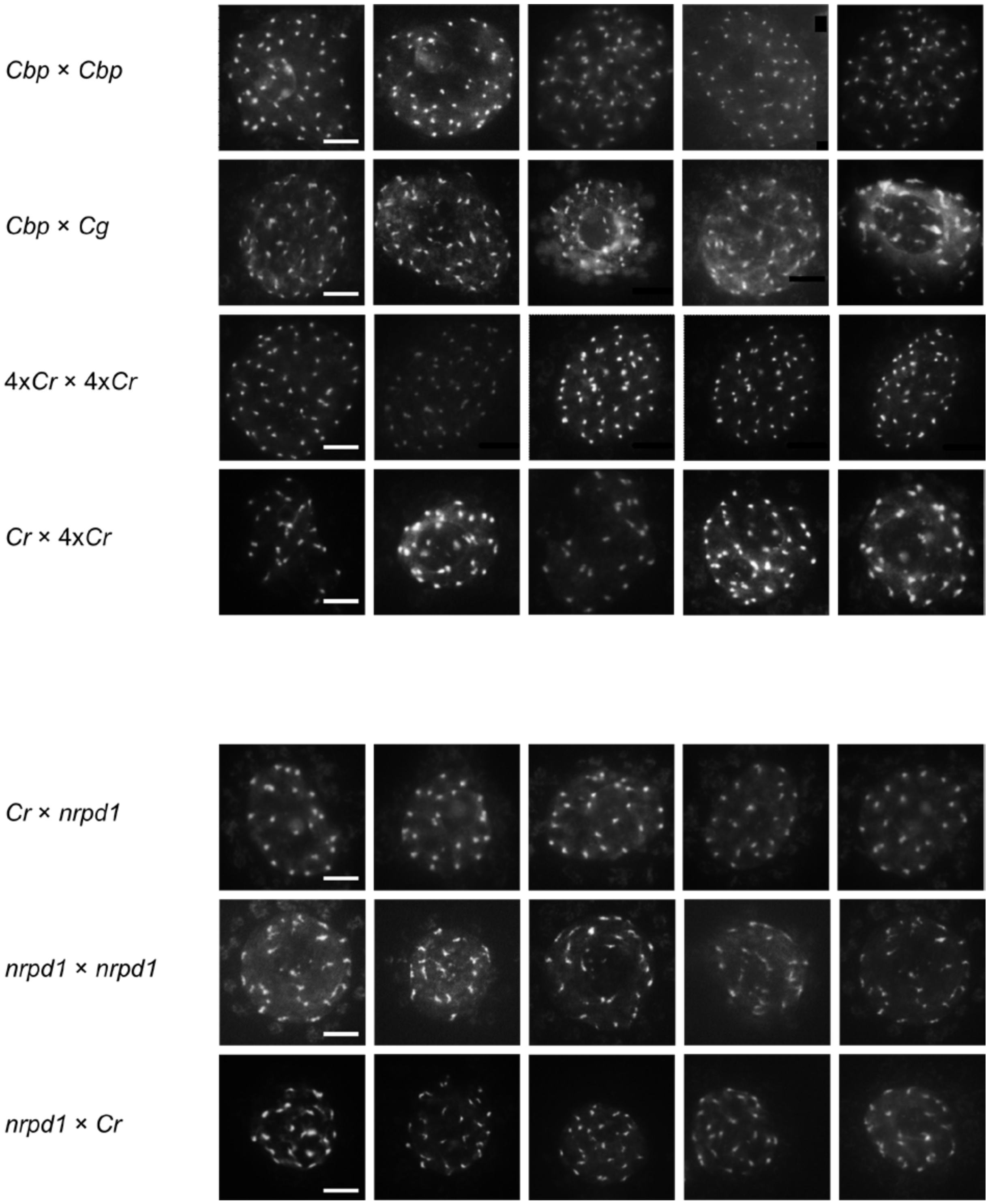

Supplementary Fig. 2: Chromatin condensation of endosperm nuclei in different *Capsella* genotypes.

Representative pictures of DAPI stained chromocenters of endosperm nuclei from different *Capsella* genotypes. Scale bar, 5 µm.

Sample name	n of raw reads	n of reads after trimming (18-25 nt)	n of reads mapped
Cr × *Cr* rep1	17.882.194	3.242.066	1.514.917
Cr × *Cr* rep2	17.293.819	3.706.363	1.864.814
Cg × *Cg* rep1	13.022.276	5.095.890	2.269.771
Cg × *Cg* rep2	22.012.779	7.026.730	2.969.020
Cr × *Cg* rep1	11.570.623	1.502.551	615.620
Cr × *Cg* rep2	13.907.822	1.730.318	747.007
nrpd1 × *Cr* rep1	30.548.500	3.830.948	1.359.792
nrpd1 × *Cr* rep2	22.019.642	2.065.888	568.481
nrpd1 × *nrpd1* rep1	19.746.933	2.101.384	528.711
nrpd1 × *nrpd1* rep2	16.966.209	1.699.865	424.553
Cg × *Cr* rep1	8.012.947	2.281.701	895.835
Cg × *Cr* rep2	11.693.785	5.552.618	2.073.302
4x*Cr* × *Cg* rep2	8.249.033	2.624.717	1.096.844
4x*Cr* × *Cg* rep2	10.294.948	3.615.278	1.682.587

Supplementary Table 1. Quality of sRNA sequencing data.

Supplementary Data 1. Gene expression in all analyzed crosses.

Table shows log2 fold changes of gene expression in the endosperm of indicated crosses.

Supplementary Data 2. List of siren loci and overlapping genomic features.

Table shows position and normalized read counts over siren loci in the endosperm of the indicated genotype.

Supplementary Data 3. List of potential *trans* targets affected in the *Cr* x *Cg* hybrid.

Table shows difference in sirenRNA accumulation (Diff_RPM) >0, differences in CHH and CHG methylation (diff_CHH) >0.01 OR diff_CHG>0.05, and differences in RNA accumulation (log2 fold change >1, p-val <0.05) in *Cr* x *Cg* compared to *Cr* x *Cr*.
